# Prion Protein of Extracellular Vesicle Regulates the Progression of Colorectal Cancer

**DOI:** 10.3390/cancers13092144

**Published:** 2021-04-29

**Authors:** Chul-Won Yun, Jun-Hee Lee, Gyeongyun Go, Juhee Jeon, Sungtae Yoon, Sang-Hun Lee

**Affiliations:** 1 Medical Science Research Institute, Soonchunhyang University Seoul Hospital, Seoul 04401, Korea; skydbs113@naver.com; 2 Institute of Tissue Regeneration Engineering (ITREN), Dankook University, Cheonan 31116, Korea; junheelee@dankook.ac.kr; 3 Department of Nanobiomedical Science and BK21 PLUS NBM Global Research Center for Regenerative Medicine, Dankook University, Cheonan 31116, Korea; 4 Department of Oral Anatomy, College of Dentistry, Dankook University, Cheonan 31116, Korea; 5 Cell & Matter Institute, Dankook University, Cheonan 31116, Korea; 6 Department of Biochemistry, College of Medicine, Soonchunhyang University, Cheonan 31151, Korea; ggy0227@naver.com; 7 Department of Biochemistry, BK21FOUR Project2, College of Medicine, Soonchunhyang University, Cheonan, 31151, Korea; 8 Stembio. Ltd., Entrepreneur 306, Soonchunhyang-ro 22, Sinchang-myeon, Asan 31538, Korea; jeonj1008@gmail.com (J.J.); yoon.st@yahoo.com (S.Y.)

**Keywords:** colorectal cancer cell, cellular prion protein, exosome, drug resistance, antibody therapeutics

## Abstract

**Simple Summary:**

Cellular prion protein (PrP^C^) are overexpressed in cancers and related to cancer proliferation, invasion, metastasis, and drug resistance. The aim of our study was to investigate the role of PrP^C^-expressing exosomes regulating the colorectal cancer cells (CRC) behavior and tumor progression. We confirmed the increased sphere formation, expression of cancer initiating genes, motility, and tumor growth by hypoxic exosomes. Also, PrP^C^-expressing exosomes induced the microenvironment of metastasis via increase of endothelial permeability and angiogenic cytokine secretion. The treatment of anti-PrP^C^ and 5-fluorouracil decreased the tumor progression. Targeting PrP^C^ is an effective therapeutic strategy in cancer therapy.

**Abstract:**

Colorectal cancer (CRC) is one of the leading causes of cancer-related death due to its aggressive metastasis in later stages. Although there is a growing interest in the tumorigenic role of cellular prion protein (PrP^C^) in the process of metastasis, the precise mechanism behind the cellular communication involving prion proteins remains poorly understood. This study found that hypoxic tumor microenvironment increased the PrP^C^-expressing exosomes from CRC, and these exosomes regulate the CRC cell behavior and tumor progression depending on the expression of PrP^C^. Hypoxic exosomes from CRC cells promoted sphere formation, the expression of tumor-inducing genes, migration, invasion, and tumor growth. Furthermore, these exosomes increased endothelial permeability, migration, invasion, and angiogenic cytokine secretion. These effects were associated with PrP^C^ expression. Application of anti-PrP^C^ antibody with 5-fluorouracil significantly suppressed the CRC progression in a murine xenograft model. Taken together, these findings indicate that PrP-expressing exosomes secreted by hypoxic CRC cells are a key factor in the tumorigenic CRC-to-CRC and CRC-to-endothelial cell communication. Significance: These findings suggest that inhibiting PrP^C^ in hypoxic exosomes during chemotherapy may be an effective therapeutic strategy in colorectal cancer.

## 1. Introduction

Colorectal cancer (CRC) is the second leading cause of cancer-related deaths and the third most prevalent malignant tumor worldwide. Early diagnosis of CRC increases the 5-year survival rate by approximately 64%, but progression of metastasis decreases the survival rate to 12% [1]. Although the ideal treatment for CRC is surgical control to remove tumor and metastases [2], chemotherapy is the typical leading strategy to control CRC [3]. Recent chemotherapy includes fluoropyrimidine-based single-agent therapy, such as 5-fluorouracil (5FU), and multiple-agent therapy, including capecitabine, irinotecan, or oxaliplatin (OX) [3]. Despite the advancements in chemotherapeutic strategies, drug resistance restricts the chemotherapeutic effect by increasing the DNA repair process and drug-releasing metabolism [4,5]. Furthermore, the tumor microenvironment, hypoxia, increases drug resistance in patients with CRC [6], suggesting that novel combination therapies, such as targeted therapy and immune checkpoint inhibitor therapy, are needed to overcome CRC [3,5].

Hypoxia is a common feature of malignant tumors, which contributes to tumor angiogenesis, aggressiveness, and metastasis [7,8]. In particular, the crosstalk between cancer cells and cells is regulated by extracellular vesicles, especially exosomes, secreted from hypoxia-stimulated cancer cells [9]. Since hypoxic stress leads to significant alterations in the molecular content and function of exosomes, hypoxic tumor-derived exosomes transfer some target genes, including glucose transporter, epidermal growth factor receptor (EGFR), transfer receptors, and P-glycoprotein, to non-hypoxic cells, resulting in the internalization of receptors and clustering of oncogene/proto-oncogene-activating receptors [10]. These hypoxia-induced exosomes promote tumor angiogenesis, invasion, metastasis, and tumor immune system. Thus, novel approaches for addressing metastatic cancer must be explored to block the hypoxic exosome-mediated communication between cancer cells and cells [10,11,12,13,14].

Cellular prion protein (PrP^C^) is a cell surface glycoprotein and misfolding of PrP^C^ is associated with neurodegenerative diseases, including transmissible spongiform encephalopathy and prion diseases [15]. In tumor biology, studies have indicated that PrP^C^ plays an important role in cancer proliferation, invasion, metastasis, apoptosis, and drug resistance [16,17]. Our recent studies have shown that hypoxia increases the expression of PrP^C^ in CRC cells and that PrP^C^ regulates cancer stem cell (CSC) markers in CRC cells and tumor progression [18,19]. In particular, tissues of stage III CRC patients highly expressed PrP^C^ with Oct4-matched expression [19]. Thus, we hypothesized that tumor hypoxic conditions increase the PrP^C^-expressing exosomes secreted by drug-resistant CRC cells, which controls CRC function and tumor progression. This study aimed to investigate the effect of exosomes derived from hypoxic 5FU- and OX-resistant CRC cells on tumorigenic potential via PrP^C^ level. Furthermore, we aimed to elucidate a novel therapeutic strategy that involve the co-administration of 5FU and anti-PrP antibody for clinical application in patients with CRC.

## 2. Materials and Methods

### 2.1. Specimens of Patients with Colorectal Cancer and Normal Controls

This study and the acquisition of clinical samples were approved by the Ethics Committee of Seoul Hospital, Soonchunhyang University (IRB: SCHUH 2018-04-032-002), and informed consent was obtained from all study participants. The serum samples of CRC patients (n = 18; grade I, n = 90; grade II, n = 90; grade III) and normal controls (n = 45) were obtained from the Biobanks of Chonbuk National University Hospital, the Ajou University, and Keimyung University Dongsan Medical Center, South Korea. CRC tissue specimens (n = 288) in an FFPE block were obtained from Soonchunhyang University. Clinical information was obtained from reports and histology sections.

### 2.2. Cell, Hypoxia Precondition and Spheroid Culture of Human Colon Cancer Cell Line and S707 Cancer Stem Cells (CSCs)

A human colon cancer cell line (SNU-C5/WT), a 5FU-resistant cell (SNU-C5/5FUR), and an oxaliplatin-resistant cell (SNU-C5/OXR) were obtained from the Chosun University Research Center for Resistant Cells (Gwangju, Korea). The cells were cultured in RPMI 1640 with 10% FBS (Thermo Fisher Scientific, Waltham, MA, USA) at 37 °C, 5% CO_2_ condition. S707 human colon CSCs were provided by Prof. Steven M Lipkin from the Department of Medicine, Weill Cornell College of Medicine (New York, NY, USA) [20]. The cells were cultured in ultra-low attachment plates as spheres in DMEM/F12 medium with supplements (Thermo Fisher Scientific) at 37 °C, 5% CO_2_ condition. SNU-C5/5FUR, SNU-C5/OXR and CSCs were incubated in a modular incubator chamber (IB Science, Daejeon, Korea) maintaining a hypoxic gas mixture (2% O_2_, 5% CO_2_, and balanced N_2_) for 48 h at 37 °C. In addition, SNU-C5/5FUR and SNU-C5/OXR and human CSCs (S707) were cultured in ultra-low attachment six-well plates for spheroid formation at 37 °C, 5% CO_2_ condition. SNU-C5/5FUR and SNU-C5/OXR and CSCs were treated with exosomes from (1) normoxic, (2) hypoxic, and (3) PRNP siRNA treated conditions. Spheroids measured with a visual inverted microscope (Olympus, Tokyo, Japan).

### 2.3. Cell Culture and Characterization of Endothelial Progenitor Cells

Human umbilical vein endothelial cells (HUVECs) (purchased from Thermo Fisher Scientific) were cultured in complete EGM-2 medium (Lonza, Walkersville, MD, USA), and characterized with flow cytometry analysis using positive HUVEC marker (anti-human CD31 or PECAM-1; and negative HUVEC markers (anti-human CD45, and anti-human CD11b) purchased from BD Pharmingen, San Diego, CA, USA.

### 2.4. Cell Isolation Targeting PrP^C^ Using Magnetic Activated Cell Sorting

Cell isolation by the expression of PrP^C^ was sorted using manual magnetic activated cell sorting (MACS) according to the manufacturer’s protocol (Miltenyi Biotec, Bergisch Gladbach, Germany). Cells were incubated with human CD230 (PrP)-Biotin primary antibody, followed by a wash with MACS rinsing solution and attached to anti-Biotin 

MicroBeads secondary antibody. After another wash, a MACS LS column with an active magnetic field was used to sort and isolate the cells, which were used for flow cytometry analysis, spheroid formation, and RNA sequencing.

### 2.5. RNA Sequencing Assay for Total RNA of Sorted Colon Cancer Cells

RNA sequencing (RNA-seq) of total RNA was performed at Macrogen using a TruSeq Stranded mRNA LT Sample Prep Kit (Illumina, San Diego, CA, USA). The colon cancer cells, sorted by PrP^C^ expression, were used for the Illumina Small RNA Sequencing protocol using the NovaSeq 6000 S4 Reagent Kit. Quality control with FastQC (v0.11.7), read trimming with Trimmomatic (v0.38), and mapped with HISAT2 (v2.1.0), Bowtie2 (v2.3.4.1), and StringTie (v1.3.4d) were performed. Gene-set enrichment analysis and functional annotation were done on differentially expressed genes, using the Gene Ontology database. Morpheus software was used for heatmap analysis.

### 2.6. Flow Cytometry Analysis

Flow cytometry analysis of Oct4, Nanog, and ALDH1A1 was performed to identify the presence of cancer stem cells. A two-color flow cytometry system (BD FACS Canto II; BD, Franklin Lakes, NJ, USA) was used to examine the immunostained cells. By comparing the results with the corresponding negative controls, the percentage of stained cells was calculated. Flow cytometry with CD81 and CD63 was used to confirm exosome markers. A two-color flow cytometry system was used to investigate the immunostained exosomes. The percentage of stained exosomes was calculated by comparing the negative controls.

### 2.7. Isolation of Exosomes

Conditioned media were obtained from CRC cells (3 × 10^6^) after 48 h of incubation with serum-free media. Exosomes were isolated from conditioned media (60 mL per group) of normoxic, hypoxic, or si-PRNP pretreated hypoxic CRC cells using an exosome isolation kit (Rosetta Exosome, Seoul, Korea). First, conditioned media was precleared by differential centrifugation and concentrated using a centrifugal filter (Sigma-Aldrich, Saint Louis, MO, USA). Additionally, then, extracellular vesicle enrichment is obtained by using Solution A, B, and C. Finally, purified exosomes is collected by the usage of spin-based size-exclusion column. The protein concentration of exosomes is measured by the BCA assay. For cell treatment, 2 μg of exosomes based on protein measurement using BCA assay were added to 2 × 10^5^ cells.

### 2.8. Identification of Exosomes via Cryo-Electron Microscopy

For cryo-electron microscopy (Cryo-EM), 5 μL of exosomes were loaded on 300-mesh EM carbon grids with a hydrophilic surface and frozen using Vitrobot (Thermo Fisher Scientific) in liquid nitrogen. The grids were observed and analyzed using Talos L120C cryoTEM (Thermo Fisher Scientific), and images were recorded at 13,000 magnification.

### 2.9. Dynamic Light Scattering Analysis

The size of exosomes derived from colon cancer cells was measured using the ELSZ-1000 analyzer (Otsuka electronics, Kobe, Japan). Briefly, exosomes (10 μL) were diluted to 1:100 in PBS. The solution was measured by performing the zeta-potential and particle size analysis to confirm the presence of exosomes.

### 2.10. Western Blot Analysis

Total protein was extracted using RIPA lysis buffer (Thermo Fisher Scientific). Cell lysates (20 μg) were separated by 10% sodium dodecyl sulfate-polyacrylamide gel electrophoresis, and proteins were transferred onto polyvinylidene fluoride membranes for detection. After washing with TBST (10 mM Tris-HCl (pH 7.6), 150 mM NaCl, and 0.05% Tween-20), the membranes were blocked with 5% skim milk for 1 h and then incubated with primary antibodies specific to PrP^C^, CD81, CD63, and β-actin, followed by washing and another incubation peroxidase-conjugated secondary antibodies. Visualization of the band was done with chemiluminescence. The whole western blot figures can be found in the supplementary materials. 

### 2.11. Detection of PrP^C^ Concentration via Enzyme-Linked Immunosorbent Assay

Concentrations of PrP^C^ in serum sample (100 μL) or isolated exosomes (50 μg) were analyzed using enzyme linked immunosorbent assay (ELISA) with a commercial kit manufactured by Lifespan Biosciences, Seattle, WA, USA. PrP^C^ were quantified by its absorbance at 450 nm using a microplate reader (BMG Labtech, Ortenberg, Germany).

### 2.12. Invasion Assay

Matrigel-coated transwell cell culture chambers (8-μm pore size; Sigma-Aldrich) and serum-free RPMI-1640 or EBM-2 medium were used to assess the invasion of SNU-C5/5FUR, SNU-C5/OXR, and HUVECs. The cells were first treated with SNU-C5/5FUR or SNU-C5/OXR exosomes derived from different conditions for: hypoxic, normoxic, and/or transfected with si-PRNP, and incubated for 72 h at 37 °C, then invasion assay was performed. Cells were stained with 2% crystal violet, and invasive cells were quantified and photographed using a light microscope.

### 2.13. Morpholometric Analysis

Morphological changes in colon cancer cell lines were examined by phase-contrast microscopy (Nikon, Tokyo, Japan). The cells were cultured in 24-well plates (7000 cells/well). Cell images were obtained using phase-contrast microscopy. The average cell size was calculated from at least three different visual fields in three independent dishes using ImageJ software.

### 2.14. Wound-Healing Migration Assay

Cells were cultured up to 90% confluence under experimental conditions, and the cell layer was scratched with a pipette tip and cultured for 24 h at 37 °C. Cell images were acquired with an inverted microscope (Eclipse TE300, Nikon, Tokyo, Japan).

### 2.15. Human Angiogenesis Protein Array

A commercially available human angiogenesis antibody array (Abcam, Cambridge, UK) was used to measure the expression of angiogenesis proteins in HUVECs treated with exosomes; approximately 200 μg of total lysates protein was analyzed following the protocol.

### 2.16. Tumorigenesis in CRC Xenograft Mice Models

A mice xenograft model of CRC using BALB/c nude mouse was created with subcutaneous injection of SNU-C5/WT cells (5 × 10^6^). When the tumors reached a volume of 10 mm^3^, 5FU (Sigma-Aldrich) and anti-PrP antibody (Santa Cruz Biotechnology) were administered together. After 28 days of drug administration, the mice were euthanized for histology. Two perpendicular tumor dimensions (a = length, b = width) were measured with a Vernier caliper and the volume (V; mm^3^) was calculated with the formula V = (a × b^2^)/2.23. The tumor specimens were fixed in 4% formaldehyde, embedded in paraffin, sliced into 4-μm-thick sections, and stained for immunohistochemistry-based analysis.

### 2.17. In Vivo Vascular Permeability Assay

For the in vivo vascular permeability assay, the mice were treated with experimental exosomes for 21 days (2 μg exosomes per injection; two injections per week). An amount of 100 mg/kg rhodamine-dextran (Sigma-Aldrich; average MW ~70,000) was administered intravenously via tail vein. Transcardiac perfusion was performed 3 h post injection to remove the excess dye. Tissues specimens were either embedded in Tissue-Plus OCT Compound (Thermo Fisher Scientific) and cryo-sectioned for fluorescent microscopy or fixed for hematoxylin and eosin (H&E) staining for histological analysis.

### 2.18. Immunofluorescence Staining

Paraffin-embeded sections of tissue samples were incubated with the primary antibodies against HSPA1L and HIF-1α (Santa Cruz Biotechnology) or against CD31, zonula occludens-1 (ZO-1), KI67, and cleaved caspase-3 (Santa Cruz Biotechnology) depending on the experimental conditions. Alexa488-conjugated or Alexa594-conjugated secondary antibodies (Thermo Fisher Scientific) were used. DAPI (Vector Laboratories, Burlingame, CA, USA) was used to mark cell nuclei. Confocal microscopy (Leica, Wetzlar, Hesse, Germany) images were taken for subsequent analysis.

### 2.19. Measurements of Oxygen Consumption Rate

The mitochondrial oxygen consumption rate (OCR) was measured using an XF96e Extracellular Flux Analyzer (Agilent Technologies, Santa Clara, CA, USA) following the manufacturer’s instructions. Sequential injections of oligomycin, carbonyl cyanide m-chlorophenylhydrazone, rotenone, and antimycin A provide information for mitochondrial basal respiration, maximal respiration, ATP turnover, and spare respiratory capacity, respectively. Final results were presented as the percentage of change compared with the control.

### 2.20. Ethics Statement

All animal studies were approved by the Institutional Animal Care and Use Committee of Soonchunhyang University and fulfilled in accordance with the National Research Council Guidelines for the Care and Use of Laboratory Animals. This study used male Balb/C nude mice (8–10 weeks old; Biogenomics, Seoul, Korea). All animals were maintained in a pathogen-free facility under a 12-h light/dark cycle at 25 °C with free access to water and laboratory chow.

### 2.21. Statistical Analysis

Results were expressed as the mean ± SEM, and a two-tailed Student’s t test or one- or two-way analysis of variance was used to compute the significance between the groups. Comparisons of three or more groups were performed using Dunnett’s or Tukey’s post hoc test. A *p* value of <0.05 was considered significant.

## 3. Results

### 3.1. Cancer Stem Cell Properties of CRC Are Linked to PrP^C^ Expression

We previously demonstrated that PrP^C^ controls cancer stem cell markers in CRC cells [19]. To investigate whether the cancer stem cell properties of CRC are linked to PrP^C^ expression, we assessed the clinicopathological features in patients (N = 288) with CRC depending on the expression of PrP^C^ (Table S1). The PrP^C^ expression was increased in 155/288 (53.8%) CRC tissue samples. The relationship between the PrP^C^ level and the clinicopathological features of the 288 CRC patients is presented in Table S1. PrP^C^ was not correlated with patient age, gender, pT stage, stage, vascular invasion, or perineuronal invasion. However, PrP^C^ expression was associated with pN stage, metastasis and lymphatic invasion (*p* = 0.014, *p* = 0.003 and *p* = 0.024, respectively). In addition, parameters of the 288 CRC patients are presented in Table S2. Although the expression of PrP^C^ was not associated with patient age, sex, pT stage, and vascular invasion, the expression of PrP^C^ was significantly correlated with pN stage (*p* = 0.046), metastasis (*p* = 0.046), stage (*p* = 0.005), lymphatic invasion (*p* = 0.002), and perineuronal invasion (*p* = 0.009) (Table S2). In serum samples of PrP^C^-positive patients with CRC, the concentration of PrP^C^ was significantly increased in stage II and III (Figure S1A). In CRC patients with stage III CRC, PrP^C^ was highly expressed in colon tissues and lymph nodes (Figure S1B). In addition, PrP^C^ in serum was significantly increased in stage III patients treated with chemotherapy, compared with that in stage III patients not treated with chemotherapy (Figure S1C).

**Figure 1:**
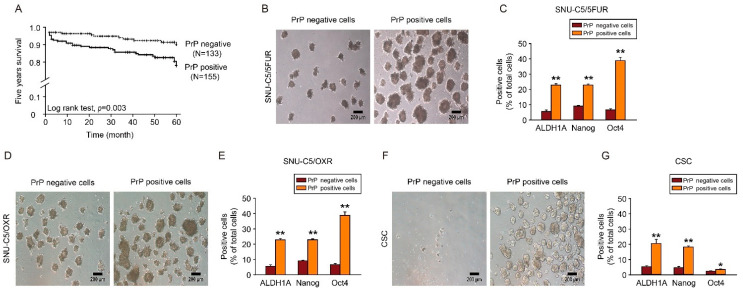
Effect of PrP^C^ of cancer stemness in CRC cells. (**A**) Five-year survival of CRC patients depending on PrP^C^ expression (negative, n = 133; positive, n = 155). (**B**–**G**) Sphere formation assay of PrP-negative and PrP-positive SNU-C5/5FUR (**B**,**C**), SNU-C5/OXR (**D**,**E**), and CSC (**F**,**G**) in ultra-low attachment plates for 2 weeks (n = 3). Cancer stem cell markers (ALDH1A, Nanog, and Oct4) were analyzed using flow cytometry analysis (n = 3). Scale bar = 200 μm. Data are represented as the mean ± SEM. * *p* < 0.05, ** *p* < 0.01 (unpaired *t*-test).

Consistent with the observed clinicopathological features in patients with CRC, the 5-year survival of PrP^C^ negative CRC patients was higher than that of PrP^C^-positive CRC patients (Figure 1A). To further explore whether PrP^C^ controls cancer stem cell properties in drug-resistant CRC cells, we investigated the formation of cancer spheres and the expression of cancer stem cell markers, including ALDH1A, Nanog, and Oct4 in 5FU-resistant CRC cells (SNU-C5/5FUR) and oxaliplatin-resistant CRC cells (SNU-C5/OXR). We found that the sphere formation capacity and cancer stem cell marker expression in each drug-resistant CRC cell were drastically enhanced in PrP-positive cells (Figure 1B–E and Figure S2A–F). Moreover, the cancer stem cell properties were significantly increased in PrP-positive CRC stem cells (CSCs) (Figure 1F,G and Figures S2G–I).

**Figure 2:**
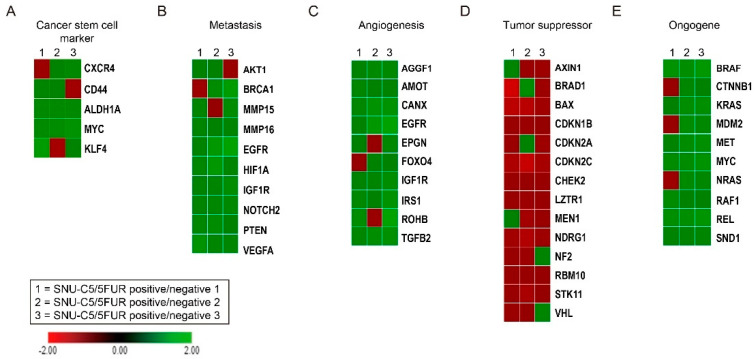
Gene analysis of CRC cells by the PrP^C^ expression. RNA-seq analysis of PrP-positive and PrP-negative SNU-C5/5FUR (n = 3). RNA-seq analysis of fold changes in PrP-positive vs. PrP-negative cells was categorized as a cancer stem cell marker (**A**), metastasis (**B**), angiogenesis (**C**), tumor suppressor (**D**), and oncogene (**E**).

To determine the effect of PrP^C^ on oncogene expression in drug-resistant CRC cells, we performed RNA sequencing on PrP-positive and PrP-negative SNU-C5/5FUR (Figure S3A–E). The transcriptome data showed that tumor progression-mediated genes, such as cancer stem cell markers, metastasis, angiogenesis, and oncogenes, were overexpressed in PrP-positive SNU-C5/5FUR, whereas tumor suppressor genes were decreased (Figure 2A–E and Figure S4). These data suggest that the PrP^C^ expression level is strongly associated with CRC progression and prognosis through regulation of cancer stem cell properties in CRC.

### 3.2. Hypoxia-Induced Exosomes Isolated from Drug-Resistant Crcs Increase Sphere Formation, Invasion, Migration, and Proliferation via Upregulation of PrP^C^

**Figure 3:**
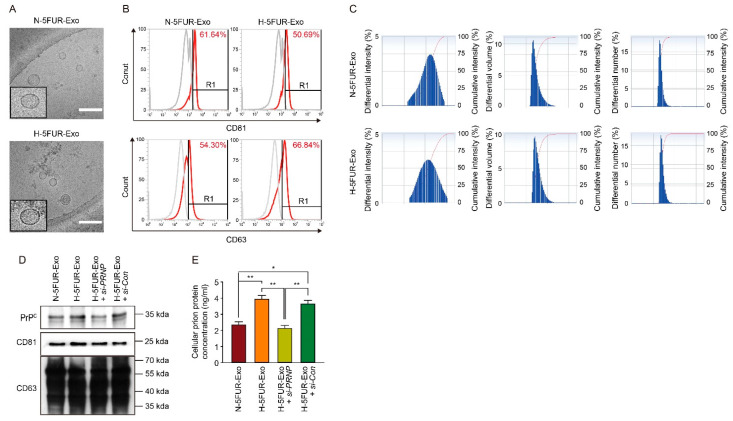
Characterization of exosomes secreted by normoxic and hypoxic drug-resistant CRC cells. (**A**) Representative cryo-electron microscopy analysis of exosomes isolated from SNU-C5/5FUR under normoxic and hypoxic conditions (n = 3). Scale bar = 200 nm. (**B**) Representative flow cytometry histogram of exosome markers, CD81 and CD63, on exosomes under normoxic and hypoxic conditions for 48 h (n = 3). (**C**) Size distribution analysis by dynamic light scattering (n = 3). (**D**) Expression of PrP^C^, CD81, and CD63 in N-5FUR-Exo, H-5FUR-Exo, H-5FUR-Exo + *si-PRNP*, and H-5FUR-Exo + *si-Con* (n = 3). (**E**) ELISA analysis of PrP^C^ expression in N-5FUR-Exo, H-5FUR-Exo, H-5FUR-Exo + *si-PRNP*, and H-5FUR-Exo + *si-Con* (n = 3). Data are represented as the mean ± SEM. * *p* < 0.05, ** *p* < 0.01 (ANOVA).

During the rapid development of tumors, hypoxia stimulates the hypersecretion of membrane-bound vesicle known as exosomes that can induce angiogenesis, metastasis, and immunosuppression to drive tumor progression [21,22]. To assess the effect of hypoxia on the component of exosomes in drug-resistant CRCs and to identify the key molecules in regulating drug-resistant CRC properties, we isolated the exosomes from SNU-C5/5FUR under normal (N-5FUR-Exo) and hypoxic conditions (H-5FUR-Exo) and characterized them (Figure 3A–C). N-5FUR-Exo and H-5FUR-Exo expressed the exosome markers CD81 and CD63 (Figure 3D). Our previous studies have shown that Oct4 and PrP^C^ are highly expressed simultaneously in patients with CRCs, and hypoxia significantly increased the level of PrP^C^ [18,19], and this was confirmed as significant PrP^C^ upregulation in N-5FUR-Exo (Figure 3D) and even higher expression H-5FUR-Exo (Figure 3D,E).

To confirm the effect of exosomes on tumor function in CRC, we initially assessed the capacity of sphere formation in CSCs after treatment with CSC-derived exosomes (Figure S5A). The number and size of spheres in CSCs were significantly increased after treatment with H-CSC-Exo, compared with that without treatment and in N-CSC-Exo (Figure S5B). In particular, the inhibition of PrP^C^ significantly decreased the number and size of spheres in CSCs treated with H-CSC-Exo (Figure S5A–C), suggesting that PrP^C^ in cancer cell-derived exosomes regulates the ability of sphere formation in CRC. In several types of CRC cells, the expression of PrP^C^ was drastically increased in SNU-C5/5FUR, SNU-C5/OXR, and CSC, compared with that in wild-type CRC cells (Figure S6A).

**Figure 4:**
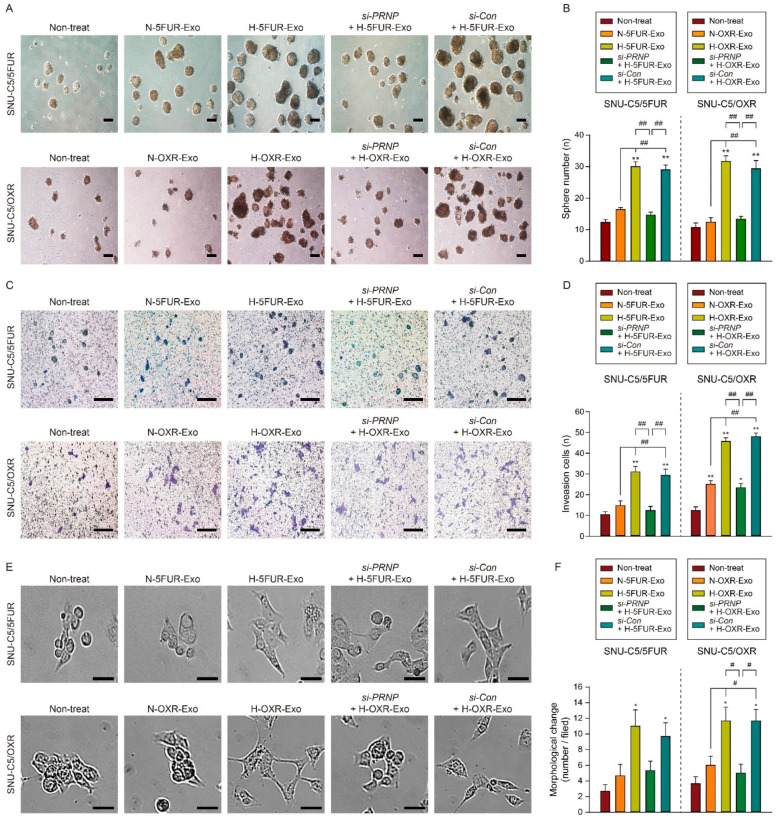
Effect of exosomes secreted by hypoxic 5FU-resistant CRC cells on tumorigenesis. (**A**) Sphere formation assay of SNU-C5/5FUR and SNU-C5/OXR treated with exosomes isolated from SNU-C5/5FUR and SNU-C5/OXR under normoxic and hypoxic conditions. Scale bar = 200 μm. (**B**) Quantification of the number of spheres is shown as a bar graph (n = 3). (**C**) Representative invasion analysis is shown as a scale bar = 200 μm. (**D**) The average number of invasive cells is shown as a bar graph (n = 3). (**E**) Representative morphological change analysis of SNU-C5/5FUR and SNU-C5/OXR treated with exosomes isolated from SNU-C5/5FUR and SNU-C5/OXR under normoxic and hypoxic conditions. Scale bar = 50 μm. (**F**) The average number of morphologically changed cells is shown as a bar graph (n = 3). Data are presented as the mean ± SEM. * *p* < 0.05, ** *p* < 0.01 vs. non-treatment; ^#^ *p* < 0.05, ^##^ *p* < 0.01 (ANOVA).

To further determine whether PrP^C^ in exosomes regulates the function of drug-resistant CRC cells, we assessed the sphere formation in SNU-C5/5FUR and SNU-C5/OXR after treatment with exosomes isolated from each cell under normoxic or hypoxic conditions (Figure 4A). Similar to CSC results, the capacity of sphere formation in SNU-C5/5FUR and SNU-C5/OXR was significantly increased following treatment with H-5FUR-Exo or H-OXR-Exo (Figure 4B and Figure S6B,C). In addition, invasion and migration capacities of drug-resistant CRC cells were significantly increased after treatment with H-5FUR-Exo or H-OXR-Exo (Figure 4C,D and Figure S7A–D). The proliferation capacity of CRC cells also significantly increased after treatment with hypoxia-stimulated exosomes (Figure S8A–F). Furthermore, the elongated mesenchymal-like morphology was significantly induced after treatment with H-5FUR-Exo or H-OXR-Exo (Figure 4E,F). However, the knockdown of PRNP blocked the effect of hypoxia-stimulated exosomes (Figure 4A–F and Figure S5A–8F). These findings indicated that increased levels of PrP^C^ in hypoxia-stimulated exosomes enhance CRC cell functions, such as sphere formation, invasion, migration, and proliferation.

### 3.3. Hypoxia-Stimulated CRC Exosomes Promote Tumor Angiogenesis and Vascular Permeability

**Figure 5:**
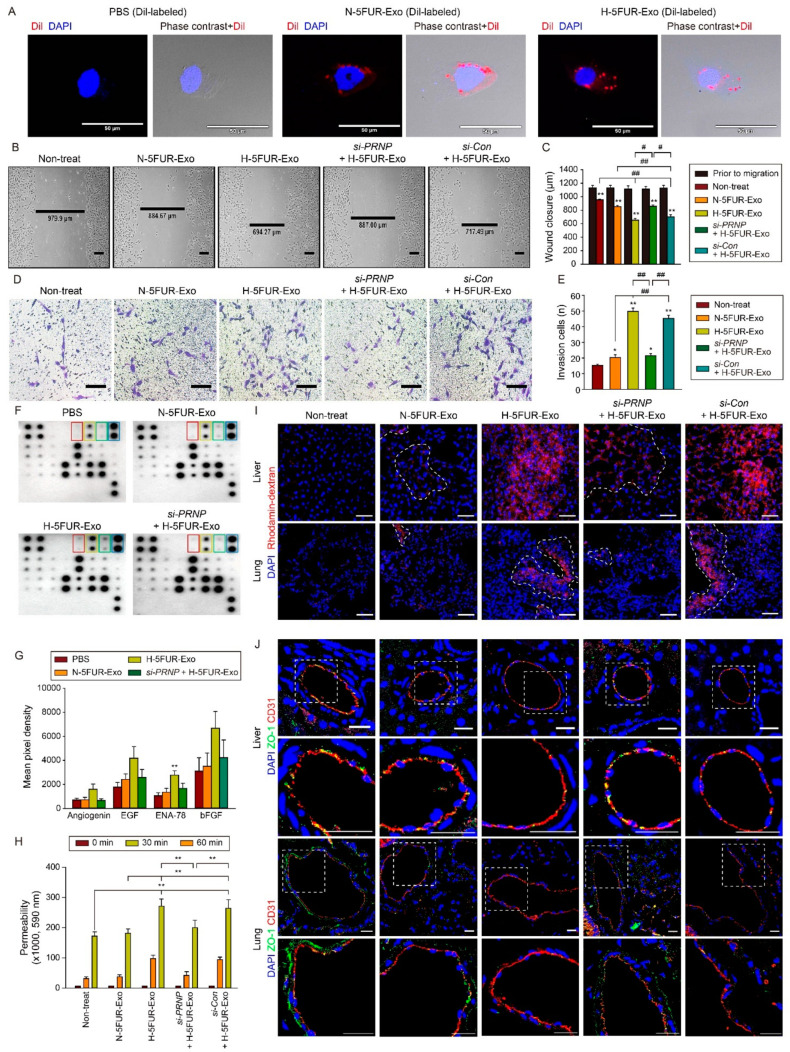
Uptake of exosomes into HUVECs and their effect on HUVECs and blood vessels. (**A**) Representative immunofluorescence analysis of intracellular uptake of DiI-labeled exosomes (red) to HUVECs. Scale bar = 50 μm (n = 3). (**B**) Representative wound healing assay of HUVECs treated with exosomes isolated from SNU-C5/5FUR under normoxic and hypoxic conditions. Scale bar = 200 μm. (**C**) The average number of wound closures is shown as a bar graph (n = 3). Data are presented as the mean ± SEM. ** *p* < 0.01 vs. Prior to migration. ^#^ *p* < 0.05, ^##^ *p* < 0.01 (ANOVA). (**D**) Representative invasion analysis of HUVECs treated with exosomes isolated from SNU-C5/5FUR under normoxic and hypoxic conditions. Scale bar = 200 μm. (**E**) The average number of invasive cells is shown as a bar graph (n = 3). Data are presented as the mean ± SEM. * *p* < 0.05, ** *p* < 0.01 vs. non-treatment. ^##^ *p* < 0.01 (ANOVA). (**F**) Representative immunoblot analysis of angiogenesis related proteins in HUVECs treated with PBS, N-5FUR-Exo, H-5FUR-Exo, and *si-PRNP* + H-5FUR-Exo (red square: angiogenin, yellow square: EGF, green square: ENA-78, and blue square: bFGF). (**G**) Average pixel densities of immunoblots are shown as the bar graph (n = 2). Data are presented as the mean ± SEM. * *p* < 0.05, ** *p* < 0.01 vs. PBS (ANOVA). (**H**) The permeability of treated HUVEC monolayers grown on 0.4-mm filters was measured by the appearance of rhodamine-dextran, which was added to the top well at the beginning of the experiment and in the bottom well during a 1-h time course. The absorbance at 590 nm at each time point is indicated (n = 3). Data are presented as the mean ± SEM. ** *p* < 0.01. (**I**) Representative images of vascular permeability in vivo on liver and lung treated with intravenously injected exosomes detected by the appearance of intravenously injected rhodamine-dextran (red) (n = 3). Scale bar = 50 μm. (**J**) Representative immunofluorescence analysis of ZO-1 (green) and CD31 (red) expression in liver and lung treated with intravenously injected exosomes. Scale bar = 50 μm.

Among the SNU-C5/5FUR, SNU-C5/OXR, and CSC, the PrP^C^ level was the highest in SNU-C5/5FUR. Additionally, we have been doing a lot of research on SNU-C5/5FUR from our previous studies [18,19,23]; we continued to carry out follow-up studies on SNU-C5/5FUR. To investigate whether hypoxia-stimulated CRC exosomes induce tumor angiogenesis, we characterized HUVECs (Figure S9A,B) and treated Dil-labeled 5FUR-exosomes with HUVECs. Fluorescence microscopic images showed that Dil-labeled 5FUR exosomes were taken up by HUVECs (Figure 5A). In HUVECs containing Dil-labeled 5FUR exosomes, H-5FUR-Exo significantly increased the level of PrP^C^ compared with N-5FUR-Exo and *si-PRNP* + H-5FUR-Exo (Figure S9C), suggesting that PrP^C^ in exosomes isolated from CRC cells is transferred into endothelial cells.

To assess the effect of hypoxia-stimulated exosomes on HUVEC migration and invasion, the migration and invasion capacities of HUVECs after treatment with exosomes were assessed. The migration and invasion capacities of HUVECs were significantly augmented after treatment with H-5FUR-Exo compared with that in other groups (Figure 5B–E). The silencing of PRNP in H-5FUR-Exo significantly inhibited the migration and invasion capacities (Figure 5B–E). In particular, the expression of angiogenic cytokine, C-X-C motif chemokine 5 (ENA-78), in HUVECs treated with exosomes significantly increased after treatment with H-5FUR-Exo (Figure 5F,G). Moreover, permeability assay showed that treatment of HUVECs with H-5FUR-Exo significantly increased the permeability of HUVECs (Figure 5H). To further reveal whether exosomes secreted by hypoxia-stimulated drug-resistant CRC cells attenuated the endothelial barrier in vivo, we injected exosomes isolated from SNU-C5/5FUR to mouse models and analyzed the permeability of blood vessels (Figure 5I). In the liver and lungs, injection with H-5FUR-Exo increased the permeability of blood vessels (Figure 5I). Furthermore, the endothelial tight junction was significantly decreased after injection of H-5FUR-Exo by decrease in the level of ZO-1, which is a tight junction protein (Figure 5J). However, knockdown of PRNP blocked the effect of H-5FUR-Exo on angiogenesis and permeability of endothelial cells in vitro and in vivo (Figure 5B–J). These data indicated that exosomes secreted by hypoxia-stimulated drug-resistant CRC cells increase tumor angiogenesis and the permeability of endothelial cells via PrP^C^.

### 3.4. Co-Administration of 5FU and Anti-PrP Antibody Inhibits CRC Progression through Suppression of PrP^C^ Level

**Figure 6:**
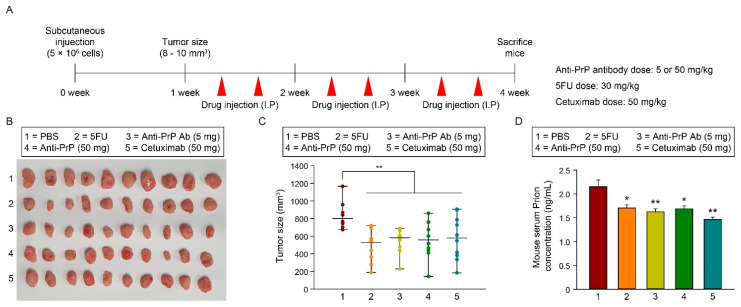
The effect of anti-PrP antibody in a CRC xenograft model. (**A**) Schematic illustration of SNU-C5/WT cells in vivo subcutaneously transplantation and injection with PBS, 5FU, anti-PrP antibodies (5 mg or 50 mg), and cetuximab (50 mg). (**B**) Photographs of tumor growth in a murine xenograft mouse model. (**C**) Quantification of tumor size in each group (n = 10). (**D**) ELISA analysis of PrP^C^ expression in sera isolated from a murine xenograft model (n = 10). Data are presented as the mean ± SEM. * *p* < 0.05, ** *p* < 0.01 (ANOVA).

To confirm the effect of 5FU, anti-PrP antibody, or cetuximab on CRC progression, we initially assessed the tumor size after treatment with 5FU, anti-PrP antibody (5 or 50 mg), or cetuximab (50 mg/kg) twice a week in an SNU-C5/WT xenograft model and investigated the level of PrP^C^ in serum (Figure 6A–D). In a wild-type CRC xenograft model, treatment with 5FU, anti-PrP antibody (5 or 50 mg), or cetuximab significantly decreased the tumor size (Figure 6B,C). The level of PrP^C^ in serum was significantly reduced after treatment with 5FU, anti-PrP antibody (5 or 50 mg), or cetuximab, compared with that treated with PBS (Figure 6D).

**Figure 7:**
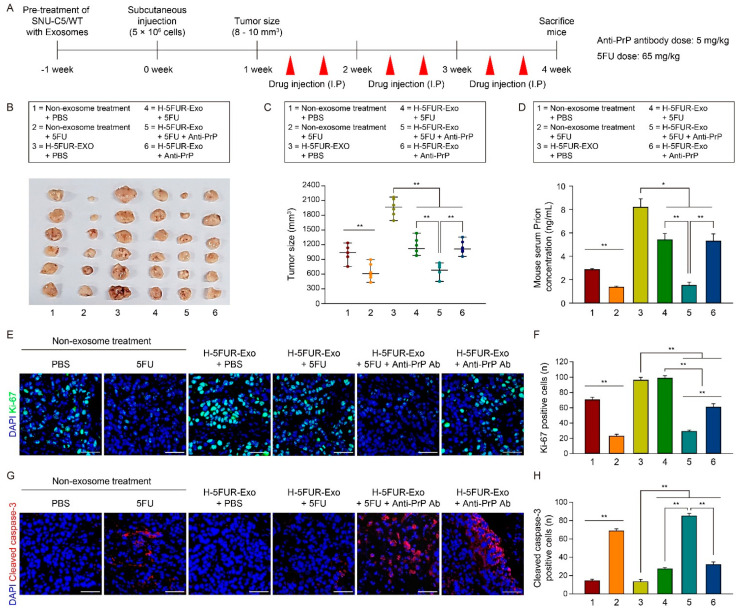
Effect of co-administration of anti-PrP antibody and 5FU in a murine xenograft model treated with exosomes secreted by hypoxic CRC cells. (**A**) Schematic illustration of SNU-C5/WT cells pretreated with/without H-5FUR-Exo in vivo subcutaneous transplantation and injection with PBS, 5FU, 5FU + anti-PrP antibody, and anti-PrP antibody. (**B**) Photographs of tumor growth in a murine xenograft mouse model. (**C**) Quantification of tumor size in each group (n = 6). (**D**) ELISA analysis of PrP^C^ expression in sera isolated from a murine xenograft model (n = 6). (**E**) Representative immunofluorescence staining analysis of Ki-67 (green) in colorectal cancer tissues. Scale bar = 50 μm. (**F**) The graph shows Ki-67-positive cells in tumor tissues (n = 3) (**G**) Representative immunofluorescence staining analysis of cleaved caspase-3 (red) in colorectal cancer tissues. Scale bar = 50 μm. (**H**) The graph shows cleaved caspase-3-positive cells in tumor tissues (n = 3). Data are presented as the mean ± SEM. * *p* < 0.05, ** *p* < 0.01 (ANOVA).

To further determine whether hypoxia-induced exosomes isolated from drug-resistant CRCs affect CRC progression and co-administration of 5FU and anti-PrP antibody suppresses CRC progression in SNU-C5/WT cells pretreated with an exosomes xenograft model (Figure 7A), we assessed the tumor size in SNU-C5/WT cells pretreated with an exosomes xenograft model after treatment with H-5FUR-Exo + PBS, H-5FUR-Exo + 5FU, H-5FUR-Exo + 5FU + anti-PrP antibody, or H-5FUR-Exo + anti-PrP antibody (Figure 7B). We also assessed the tumor size in an SNU-C5/WT xenograft model, as a negative control, after treatment with PBS (no Exo + PBS) and 5FU (no Exo + 5FU) (Figure 7B). In an SNU-C5/WT pretreated with H-5FUR-Exo xenograft model, co-treatment with 5FU and anti-PrP antibody (H-5FUR-Exo + 5FU + Anti-PrP) significantly decreased the tumor size (Figure 7C). The concentration of PrP^C^ in serum was also significantly reduced after co-treatment with 5FU and anti-PrP antibody, compared with that in cells treated with PBS (H-5FUR-Exo), 5FU (H-5FUR-Exo + 5FU), or anti-PrP antibody (H-5FUR-Exo + anti-PrP) (Figure 7D). In the context of tumor proliferation in vitro, co-treatment of SNU-C5/WT with anti-PrP antibody and 5FU significantly decreased the S phase of the cell cycle (Figure S10A,B). In particular, the S phase after co-treatment with anti-PrP antibody and 5FU was decreased in a 5FU dose-dependent manner (Figure S10C). In comparison with the antiproliferative effect of the anti-PrP antibody and cetuximab, treatment with a high concentration of cetuximab (10 μg/mL) decreased the S phase of the cell cycle of SNU-C5/WT, compared with that treated with 5FU, anti-PrP antibody, or low concentration of cetuximab (1 μg/mL) (Figure S11A). Co-treatment with cetuximab, an anti-PrP antibody, and 5FU showed the most suppressive effect on CRC proliferation (Figure S11B). Furthermore, treatment of SNU-C5/WT with anti-PrP antibody or cetuximab significantly decreased mitochondrial oxidative phosphorylation, including mitochondrial basal respiration, ATP turnover, and spare respiratory capacity, compared with the non-treatment group (Figure S12A–E). In an SNU-C5/WT pretreated with H-5FUR-Exo xenograft model, co-administration of 5FU and anti-PrP antibody significantly reduced the expression of Ki-67 in tumor tissues (Figure 7E,F). Conversely, the level of apoptotic marker cleaved caspase-3 in tumor tissues was significantly increased after co-treatment with 5FU and anti-PrP (Figure 7G,H). In the H-5FUR-Exo-treated tumor tissues, immunofluorescence staining of ZO-1 showed that the tight junctions of tumor blood vessels increased after co-administration of 5FU and anti-PrP antibody (Figure S13). Furthermore, injection with anti-PrP antibody did not show pathological lesions in the brain, kidney, liver, and lung (Figure S14). These findings suggest that co-administration of 5FU and anti-PrP antibody suppresses CRC progression by blocking PrP^C^.

## 4. Discussion

It has been shown that PrP^C^ regulates proliferation, drug resistance, metastasis, and cancer stem cell properties in various type of cancers including pancreatic, breast, and colon cancers [24]. Although exosomal PrP^C^ is known to inhibit amyloid beta-mediated neurotoxicity in Alzheimer’s disease [25], and facilitate intercellular prion transmission in prion disease [26], studies on the effect of exosomal PrP^C^ on the tumor progression are limited. In this study, we demonstrated that exosomal PrP^C^ promotes proliferation, invasion and migration of cancer cells. In addition, we found that exosomal PrP^C^ inhibits migration (Figure 5B,C) and invasion (Figure 5D,E) of vascular endothelial cells and increases permeability of blood vessels (Figure 5I,J). Therefore, PrP antibody treatment can reduce permeability of blood vessels and inhibit metastatic CRC development by neutralizing exosomal PrP. In addition, we also demonstrated that co-administration of anti-PrP antibody with chemotherpy can improve cancer treatment efficacy.

This study showed that hypoxia induced the expression of PrP^C^ in exosomes secreted by drug-resistant CRC cells and that PrP^C^-expressing exosomes promote cancer stem cell properties and tumor progression. PrP^C^ is a highly ubiquitous glycoprotein that affects the process of tumor progression, such as proliferation, migration, invasion, metastasis, chemoresistance, and apoptosis, as well as stemness of cancer cells [17,27,28]. Previous studies indicated that PrP^C^ promotes tumor metastasis, epithelial–mesenchymal transition, and glucose metabolism through the regulation of Fyn, cytoskeletal regulatory proteins [29,30]. PrP^C^ also regulates multi-drug resistance via interaction with CD44 [31,32]. Under hypoxic conditions, PrP^C^ induces tumor progression in CRC by targeting the heat shock protein 70 member 1-like (HSPA1L)/HIF-1α/GP78 signal axis [18]. Our previous data have shown a correlation between high PrP^C^ expression and clinicopathological features in patients with CRC, including metastasis risk, advanced clinical stage, and survival of CRC patients [18]. The expression of PrP^C^ in tumor tissues from stage III CRC patients is matched with Oct4 expression, indicating that co-expression of PrP^C^ and Oct4 is involved in CRC metastasis [19]. This study revealed that PrP-positive cells were increased in the properties of a cancer stem cell. In RNA-seq data, gene expressions associated with cancer stem cell markers, metastasis, angiogenesis, and oncogenes were significantly increased in PrP-positive cells, whereas tumor suppressor genes were reduced. Exosomes secreted by hypoxic drug-resistant CRC cells enhanced CRC sphere formation, invasion, migration, proliferation, and tumor progression through upregulation of PrP^C^ levels. These findings indicated that PrP^C^ plays a pivotal role in CRC behavior, suggesting that PrP^C^ could be a novel CRC marker for targeted therapy in CRC patients.

Hypoxia is a pathophysiological tumor microenvironment, which affects the cell cycle, morphological conformation, energy metabolism, differentiation, apoptosis, and autophagy [10,33,34]. Under hypoxic conditions, cancer cells regulate a wide range of gene expression in conjunction with the major components of the hypoxia signaling pathway through expression of HIFs [8,10]. In patients with pancreatic tumors, the level of HIF-1α expression was significantly increased [35]. Among HIFs, the overexpression of HIF-1α in response to hypoxia promotes tumor blood vessel formation, aggressiveness, metastasis, and drug resistance. Hypoxia and HIF-1α contribute to abnormal blood vessels, which are newly formed by discontinuous endothelium and the blockage of lymphatic drainage, resulting in the production of vascular hyperpermeability and increased permeation [36,37].

Recently, several studies have revealed that the crosstalk between tumor cells and the tumor microenvironment is a key factor for tumor progression through extracellular vesicles and exosomes secreted by hypoxic cancer cells [9,10]. Hypoxia-induced secreted exosomes released from cancer cells contain plasma membrane receptors, including glucose transporter, EGFR, P-glycoprotein, and multidrug resistance protein 1; angiogenic proteins, such as VEGF, FGF, and angiogenin; and various noncoding RNAs, including miRNAs and lncRNAs [34,37,38].

Our study indicated that targeted genes associated with cancer stem cell markers, metastasis, angiogenesis, and oncogenes were significantly increased in exosomes secreted from hypoxic 5FU-resistant CRC cells. In particular, the expression of these genes in exosomes was regulated by the expression of PrP^C^. These exosomes enhanced the cancer sphere formation, invasion, migration, and proliferation in drug-resistant and cancer stem cells in CRC. In particular, exosomes secreted by hypoxic drug-resistant CRC cells are incorporated into HUVECs and increase the migration, invasion, permeability, and production of angiogenic cytokines.

In an in vivo study, these exosomes augmented the vascular hyperpermeability through inhibition of tight junction protein ZO-1 expression. These effects were blocked by silencing of *PRNP*. For stabilization of PrP^C^, our previous study showed that HIF-1α-induced HSPA1L downregulated the expression of GP78, a ubiquitinase for PrP^C^, resulting in the stabilization of PrP^C^ under hypoxic conditions [18]. In lung cancer, exosomes secreted by hypoxic cancer increased the expression of miR-23a, which inhibited the expression of ZO-1, resulting in the induction of vascular permeability and cancer trans-endothelial migration [12]. Exosomes secreted by metastatic breast cancer also destroy endothelial ZO-1 expression and integrity through the upregulation of miR-105 [39]. This evidence indicates that exosomes secreted by hypoxic tumor cells play important roles in the reduction in endothelial integrity and tumor progression, suggesting that targeting of PrP-expressing exosomes secreted by hypoxic tumors might be a novel strategy for patients with CRC.

Clinically, antibody therapy for tumors provides the possibility for treating patients with cancer in a targeted fashion by decreasing severe side effects, compared with conventional chemotherapy [40]. For the development of advanced and highly therapeutically efficient antibody therapy in cancer biology, discovery of specific molecular biomarkers in a wide range of solid malignancies is a key process for beneficial therapeutic outcomes [40]. According to the unmet medical needs of patients with tumors, tumor therapeutic antibodies, including trastuzumab (anti-human epidermal growth factor receptor 2 [HER2] therapy) and cetuximab (anti-EGFR therapy), were developed and clinically approved as treatments for a solid carcinoma and a broad range of cancers, respectively [41,42].

Cetuximab is a chimeric human mouse anti-EGFR monoclonal antibody and it is used in combination with chemotherapy or as a single agent in metastatic colon cancer and metastatic squamous cell head and neck cancer [43]. EGFR is overexpressed in most epithelial cell carcinomas such as colorectal cancer, breast cancer, and lung cancer, and it is known that activation of EGFR promotes cancer proliferation, angiogenesis, metastasis and inhibits apoptosis [44]. Cetuximab selectively binds to EGFR and competitively inhibits the binding of EGF and other ligands, preventing its activation, eventually inhibiting the growth of cancer cells and production of MMP and EGF and inducing apoptosis [45].

However, intrinsic phenotypic variation and adaptive phenotypic modifications in tumor cells can induce repeated exposure to sub-optimal doses of the biotherapeutic, resulting in acquired resistance to monoclonal antibody therapy [40,46,47]. Antibody–drug conjugates (ADC) are another option for treating tumors as a novel antibody-based therapeutic. ADC consist of targeted antibody and anti-cancer drugs covalently attached to the antibody, resulting in ADC reaching the tumor and killing the tumor. Although ADC have the potential for this concept, the clinical outcomes are limited because some chemical drugs can be released from the tumor and diffuse into the surrounding cells [40,48].

In this study, we identified a novel CRC target, PrP^C^, and assessed the effect of anti-PrP antibody on CRC by co-administration of 5FU. In vitro, anti-PrP significantly inhibited proliferation and mitochondrial respiration. In a murine exosome non-treated xenograft model, the administration of anti-PrP antibody significantly decreased the tumor size and serum PrP^C^ concentration. This effect was similar to the effect of cetuximab. In an H-5FUR-Exo-treated xenograft model, co-administration of anti-PrP antibody and 5FU significantly reduced the tumor size, PrP^C^ expression, and tumor cell proliferation. Furthermore, co-administration drastically increased the number of apoptotic CRC cells in tumor tissues. These findings indicate that co-administration of anti-PrP antibody and 5FU suppresses CRC tumor progression, suggesting that anti-PrP antibody-based therapy could be a novel and powerful strategy for clinical application in patients with CRC.

## 5. Conclusions

Taken together, these results indicate that exosomes secreted by hypoxic drug-resistant CRC cells are key regulators of CRC progression. PrP^C^ is a pivotal messenger for regulating CRC behavior through the secretion of exosomes by hypoxic tumors. Furthermore, our results suggest the possibility of clinical application of anti-PrP antibody with anti-cancer drugs. Although our results indicated that the administration of anti-PrP antibody did not have an effect on several organs and tissues in a pre-clinical study, its side effect and safety should be investigated prior to the application of this antibody in patients with CRC. In conclusion, the co-administration of anti-PrP antibody and anti-cancer drugs might be a potential therapeutic strategy for patients with CRC through inhibition of exosomal PrP^C^ expression and suppression of CRC progression.

## Data Availability

The data presented in this study are available on request from the corresponding author.

## Supplementary Materials

The following are available online at https://www.mdpi.com/article/10.3390/cancers13092144/s1, Table S1: Clinicopathological features in patients with colorectal cancer, Table S2: Cox regression analysis of the clinicopathological parameters in colorectal cancer patients, Figure S1: Identification of PrP^C^ expression in samples of CRC patients, Figure S2: Analysis of PrP^C^ fractionation, sphere number, and sphere size in drug-resistant CRC cells and CSCs, Figure S3: NGS analysis of PrP-negative and PrP-positive SNU-C5/5FUR, Figure S4: Gene analysis of CRC cells by the PrP^C^ expression, Figure S5: Sphere formation of CSCs after treatment with CSC exosomes, Figure S6: Sphere size of drug-resistant CRC cells, Figure S7: Wound healing assay of drug-resistant CRC cells after treatment with exosomes, Figure S8: Proliferation capacity of drug-resistant CRC cells after treatment with exosomes, Figure S9: Characterization of HUVECs and expression of PrP^C^ after treatment with exosomes, Figure S10: Inhibitory effect of anti-PrP antibody with 5FU on proliferation of CRC cells, Figure S11: Inhibitory effect of anti-PrP antibody with 5FU and cetuximab on proliferation of CRC cells, Figure S12: Mitochondrial respiration in CRC cells after treatment with anti-PrP antibody and cetuximab, Figure S13: Effect of anti-PrP antibody and 5FU on vascular permeability in vivo, Figure S14: Analysis of cytotoxicity of anti-PrP antibody in murine tissues.

## Abbreviations

5FU: 5-fluorouracil; CSC: cancer stem cell; CRC: colorectal cancer; EGF: epidermal growth factor; EGFR: epidermal growth factor receptor; ELISA: enzyme-linked immunosorbent assay; ENA-78: C-X-C motif chemokine 5; FGF: fibroblast growth factor; H-5FUR-Exo: exosomes from SNU-C5/5FUR under hypoxia condition; H-OXR-Exo: exosomes from SNU-C5/OXR under hypoxia condition; HIF-1α: hypoxia-inducible factor 1 alpha; HSPA1L: heat shock protein 70 member 1-like; HUVEC: human umbilical vein endothelial cell; IGF-1: insulin-like growth factor-1; MACS: magnetic activated cell sorting; N-5FUR-Exo: exosomes from SNU-C5/5FUR under normal condition; N-OXR-Exo: exosomes from SNU-C5/OXR under normal condition; NGS; next generation sequence; PrP^C^: cellular prion protein; OCR: oxygen consumption rate; OX: oxaliplatin; SNU-C5/5FUR: 5FU-resistant SNU-C5 cells; SNU-C5/OXR: oxaliplatin-resistant SNU-C5 cells; SNU-C5/WT: wild type SNU-C5 cells; VEGF: vascular endothelial growth factor; ZO-1: zonula occludens-1.
