# Correction: Yun et al. Prion Protein of Extracellular Vesicle Regulates the Progression of Colorectal Cancer. *Cancers* 2021, *13*, 2144

**DOI:** 10.3390/cancers13143560

**Published:** 2021-07-16

**Authors:** Chul-Won Yun, Jun-Hee Lee, Gyeongyun Go, Juhee Jeon, Sungtae Yoon, Sang-Hun Lee

**Affiliations:** 1 Medical Science Research Institute, Soonchunhyang University Seoul Hospital, Seoul 04401, Korea; skydbs113@naver.com; 2 Institute of Tissue Regeneration Engineering (ITREN), Dankook University, Cheonan 31116, Korea; junheelee@dankook.ac.kr; 3 Department of Nanobiomedical Science and BK21 PLUS NBM Global Research Center for Regenerative Medicine, Dankook University, Cheonan 31116, Korea; 4 Department of Oral Anatomy, College of Dentistry, Dankook University, Cheonan 31116, Korea; 5 Cell & Matter Institute, Dankook University, Cheonan 31116, Korea; 6 Department of Biochemistry, College of Medicine, Soonchunhyang University, Cheonan 31151, Korea; ggy0227@naver.com; 7 Department of Biochemistry, BK21FOUR Project2, College of Medicine, Soonchunhyang University, Cheonan 31151, Korea; 8 Stembio. Ltd., Entrepreneur 306, Soonchunhyang-ro 22, Sinchang-myeon, Asan 31538, Korea; jeonj1008@gmail.com (J.J.); yoon.st@yahoo.com (S.Y.)

The authors wish to make the following corrections to this paper [1]:

The authors are sorry to report that the paper contains errors in Figures 6 and 7, and Supplemental Figure S12 and misses the information for these figures in the results (Section 3.4) in the recently published paper [1]. Due to unintentional errors made during the preparation of the figures, figure legends, and manuscript, the authors mistakenly used the unmatched values of Anti-PrP antibody dose or cetuximab dose in Figures 6 and 7, and Supplemental Figure S12. The authors wish to replace the correct values of the Anti-PrP antibody dose or Cetuximab dose in Figures 6 and 7, and Supplemental Figure S12 and to revise the figure legends and in the results (Section 3.4). Consequently, the authors wish to make the following corrections to the paper:

In Section 2.4 “Cell Isolation Targeting PrP^C^ using Magnetic Activated Cell Sorting”, we merged the two paragraphs of the original manuscript into one paragraph. It should be replaced with the following version: 

“Cell isolation by the expression of PrP^C^ was sorted using manual magnetic activated cell sorting (MACS) according to the manufacturer’s protocol (Miltenyi Biotec, Bergisch Gladbach, Germany). Cells were incubated with human CD230 (PrP)-Biotin primary antibody, followed by a wash with MACS rinsing solution and attached to anti-Biotin MicroBeads secondary antibody. After another wash, a MACS LS column with an active magnetic field was used to sort and isolate the cells, which were used for flow cytometry analysis, spheroid formation, and RNA sequencing.”

In Section 2.16 “Tumorigenesis in CRC Xenograft Mice Models”, the formula V = (a × b^2^)/2.23 should be changed to V = (a × b^2^)/2.

In Section 2.18 “Immunofluorescence Staining”, the words “against HSPA1L and HIF-1α (Santa Cruz Biotechnology) or” were removed from the first sentence.

In Section 3.4 “Co-Administration of 5FU and Anti-PrP Antibody Inhibits CRC Progression through Suppression of PrP^C^ Level”, the first paragraph, we changed the value of Anti-PrP antibody dose from “5 or 50 mg” to “0.05 or 0.5 mg/kg”, value of cetuximab dose from “50 mg/kg” to “0.5 mg/kg”. 

The corrected Figures 6 and 7 and Supplemental Figure S12 are listed below:

**Figure 6:**
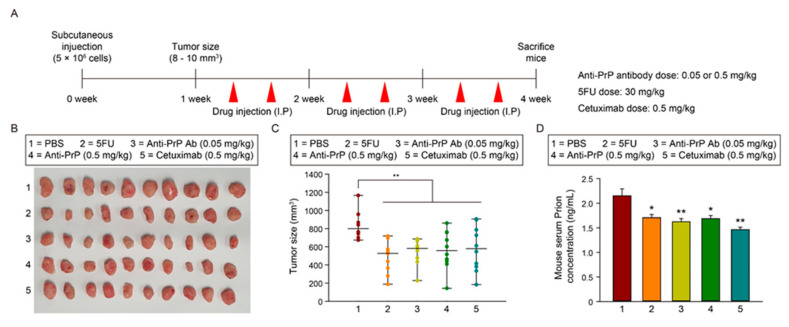
The effect of anti-PrP antibody in a CRC xenograft model. (**A**) Schematic illustration of SNU-C5/WT cells in vivo subcutaneously transplantation and injection with PBS, 5FU, anti-PrP antibodies (0.05 or 0.5 mg/kg), and cetuximab (0.5 mg/kg). (**B**) Photographs of tumor growth in a murine xenograft mouse model. (**C**) Quantification of tumor size in each group (*n* = 10). (D) ELISA analysis of PrP^C^ expression in sera isolated from a murine xenograft model (*n* = 10). Data are presented as the mean ± SEM. * *p* < 0.05, ** *p* < 0.01 (ANOVA).

**Figure 7:**
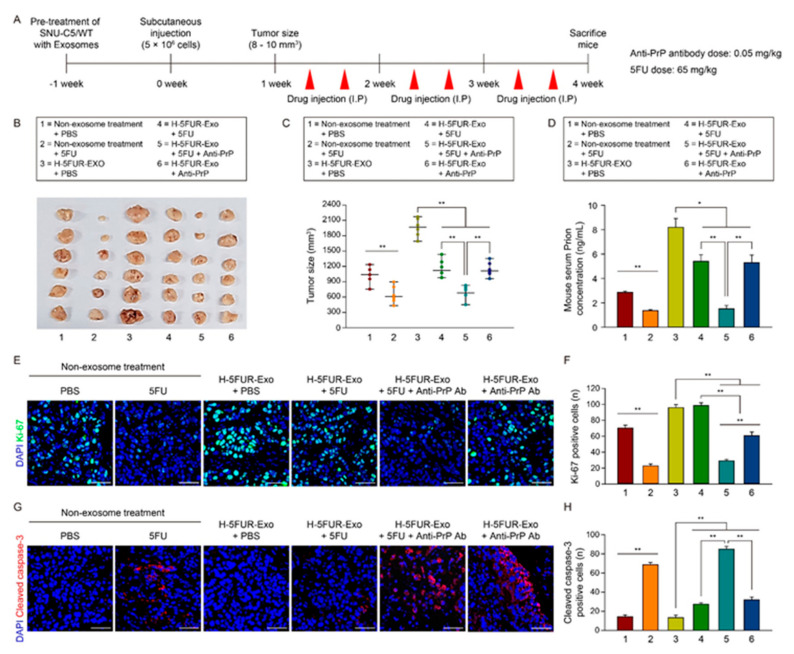
Effect of co-administration of anti-PrP antibody and 5FU in a murine xenograft model treated with exosomes secreted by hypoxic CRC cells. (**A**) Schematic illustration of SNU-C5/WT cells pretreated with/without H-5FUR-Exo in vivo subcutaneous transplantation and injection with PBS, 5FU, 5FU + anti-PrP antibody, and anti-PrP antibody. (**B**) Photographs of tumor growth in a murine xenograft mouse model. (**C**) Quantification of tumor size in each group (*n* = 6). (**D**) ELISA analysis of PrP^C^ expression in sera isolated from a murine xenograft model (*n* = 6). (**E**) Representative immunofluorescence staining analysis of Ki-67 (green) in colorectal cancer tissues. Scale bar = 50 μm. (**F**) The graph shows Ki-67-positive cells in tumor tissues (*n* = 3) (**G**) Representative immunofluorescence staining analysis of cleaved caspase-3 (red) in colorectal cancer tissues. Scale bar = 50 μm. (**H**) The graph shows cleaved caspase-3-positive cells in tumor tissues (*n* = 3). Data are presented as the mean ± SEM. * *p* < 0.05, ** *p* < 0.01 (ANOVA).

**Figure S12:**
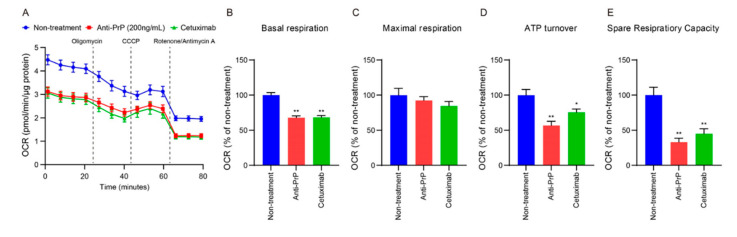
Mitochondrial respiration in CRC cells after treatment with anti-PrP antibody and cetuximab. * *p* < 0.05, ** *p* < 0.01.

The authors state that this correction does not modify the scientific results of the study. The authors would like to apologize for any inconvenience caused by this mistake.
