# Supplementary material for: Prion Protein of Extracellular Vesicle Regulates the Progression of Colorectal Cancer
Source: Cancers (Basel). 2021 Apr 29;13(9):2144. doi: 10.3390/cancers13092144 (PMC8124505; doi:10.3390/cancers13092144)

Supplemental Figure 1

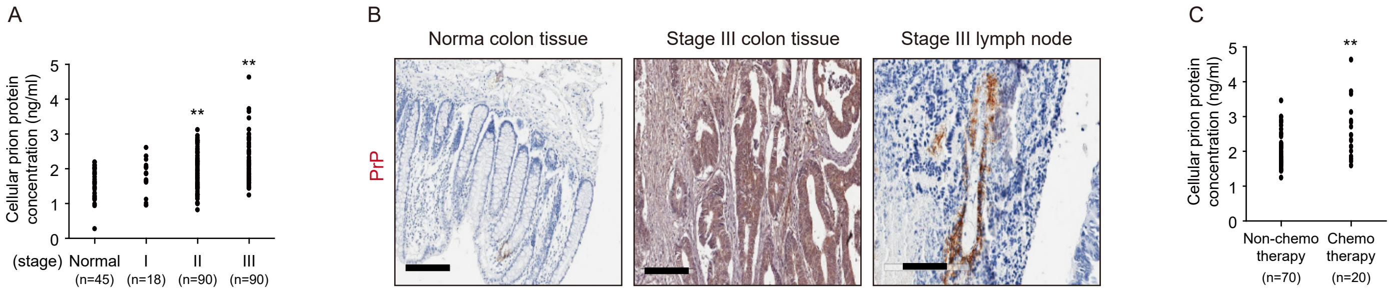

Supplemental Figure 2

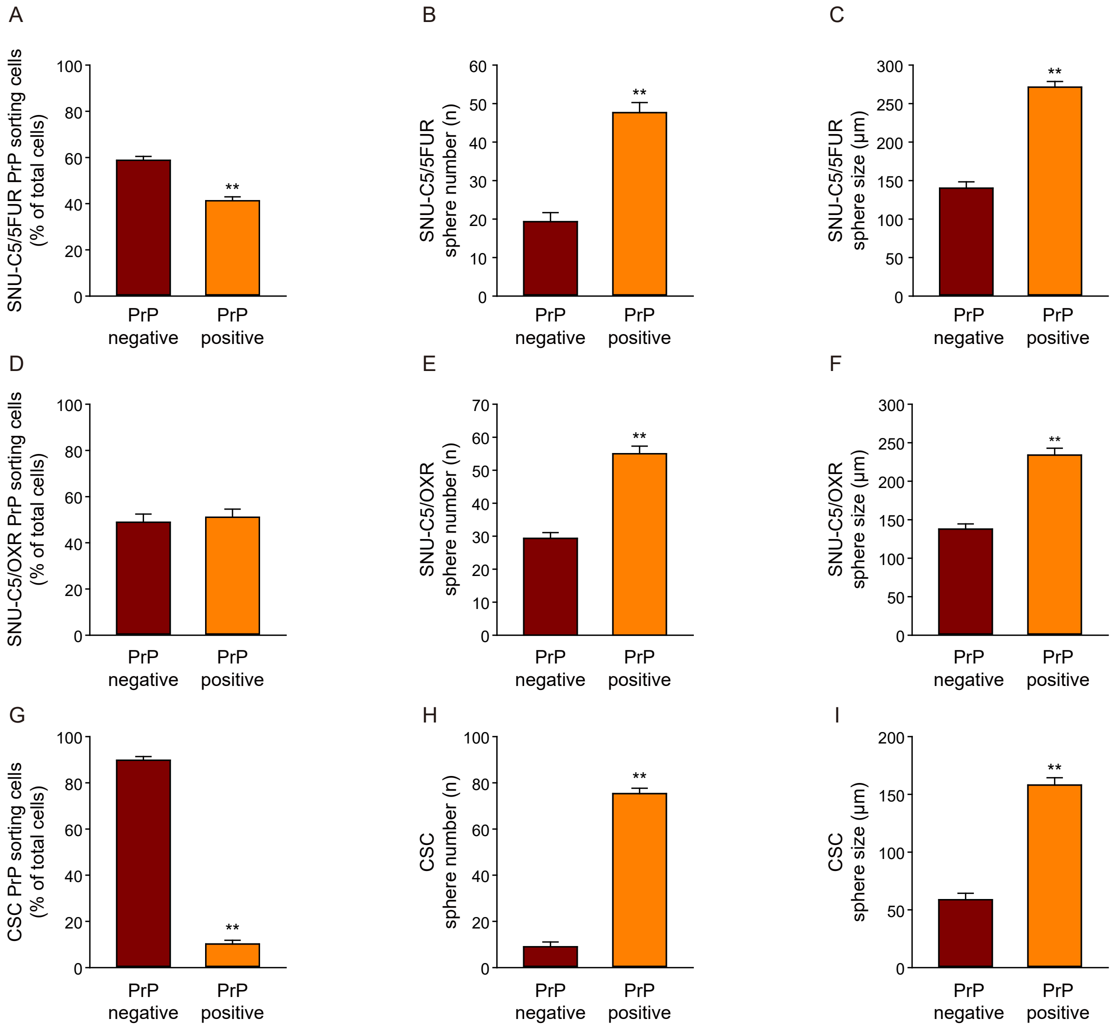

Supplemental Figure 3

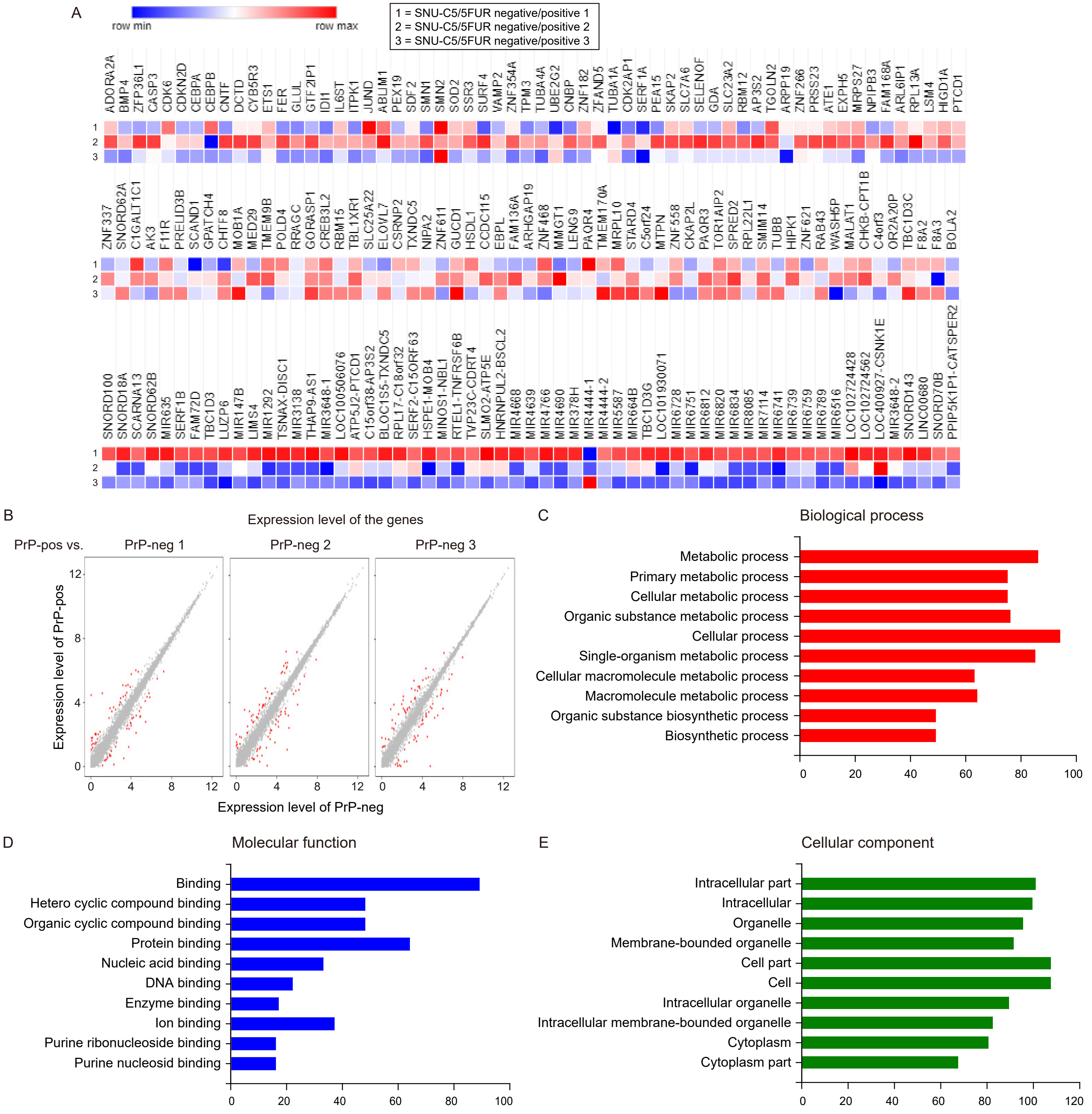

Supplemental Figure 4

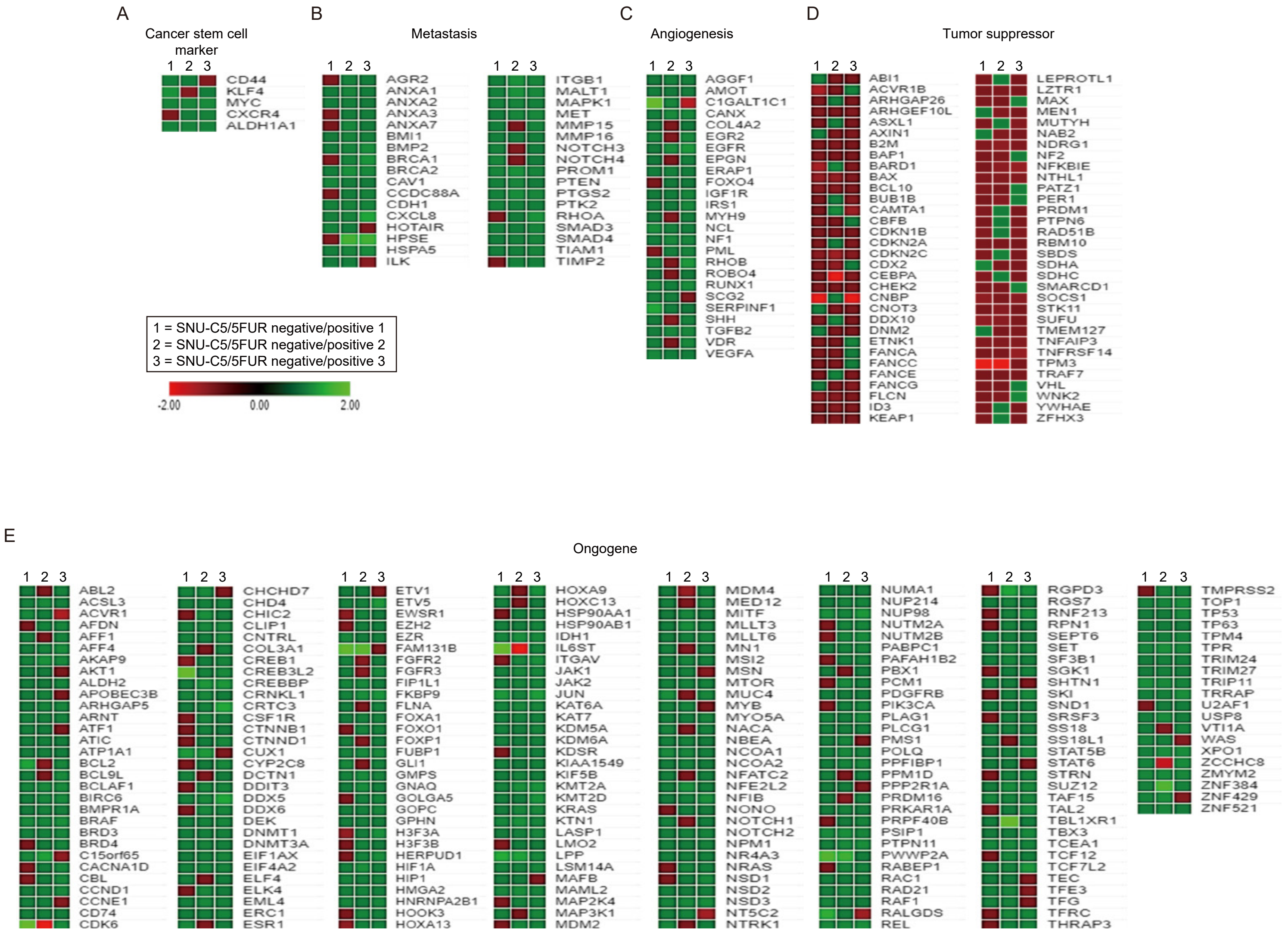

Supplemental Figure 5

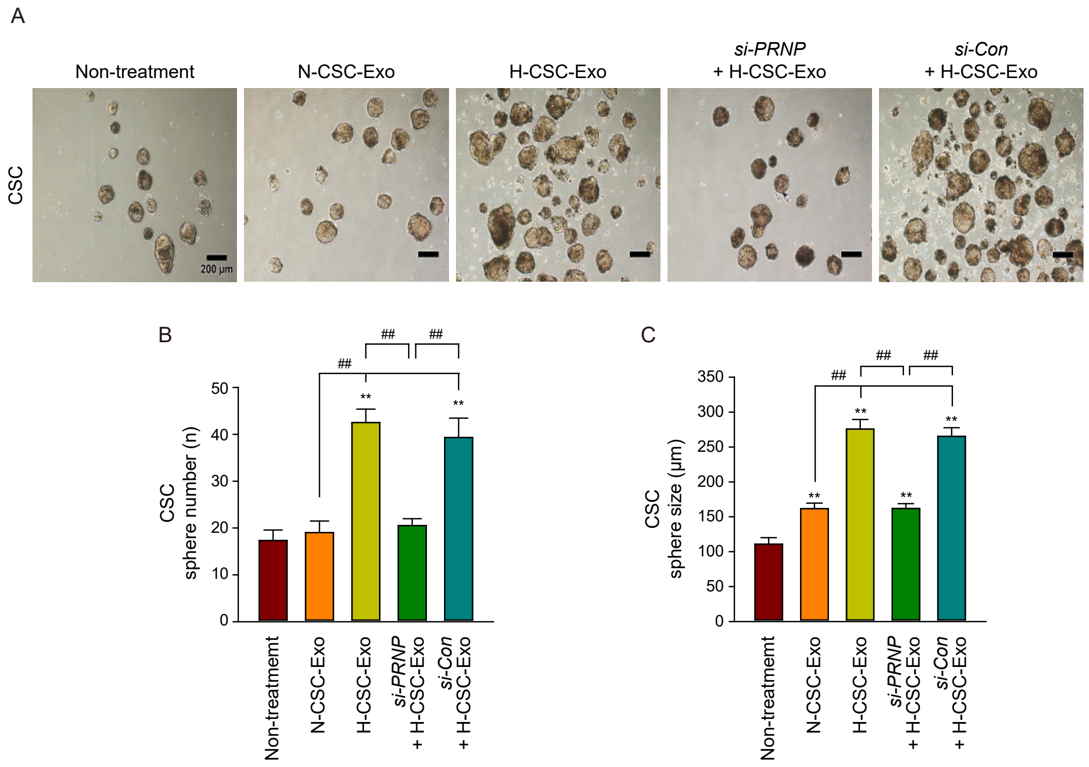

Supplemental Figure 6

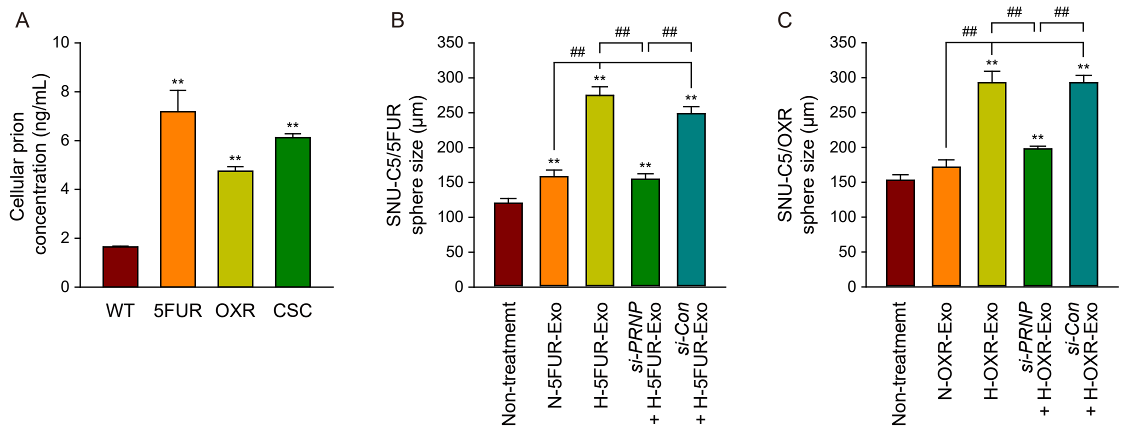

Supplemental Figure 7

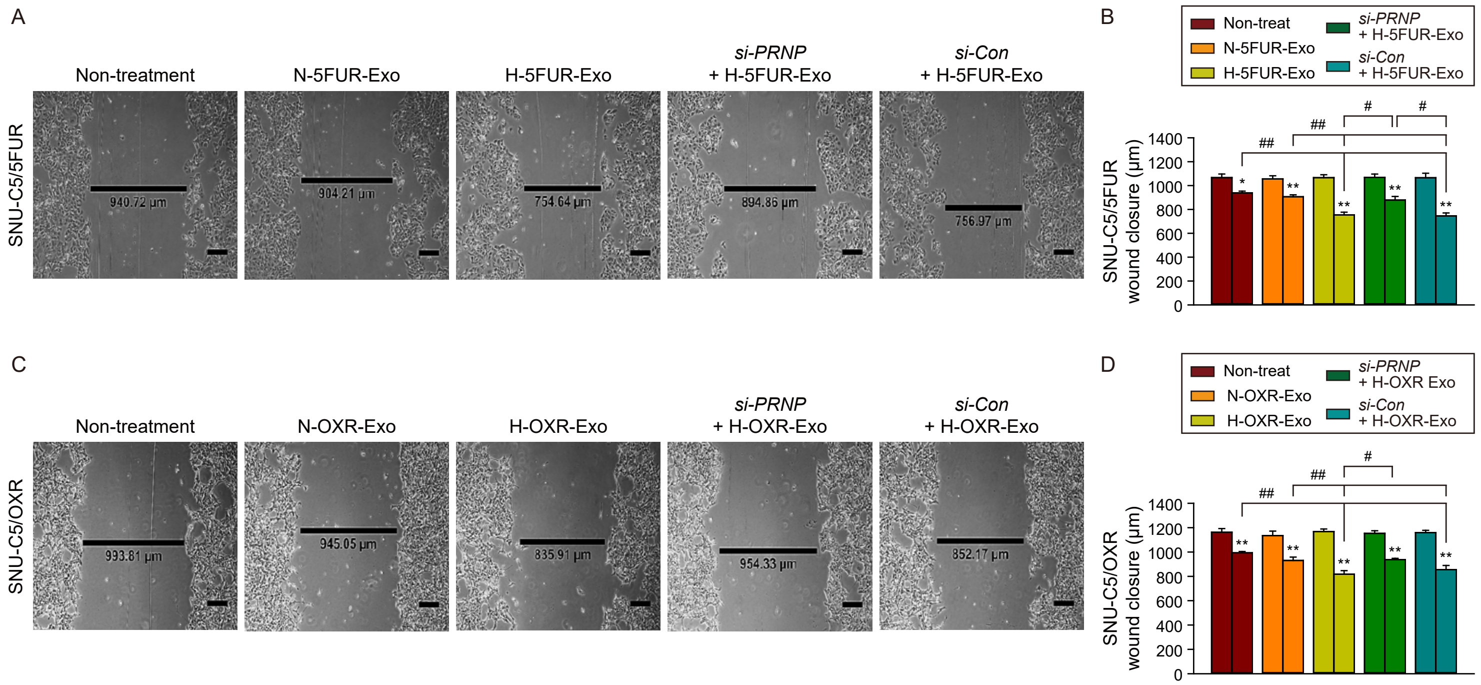

Supplemental Figure 8

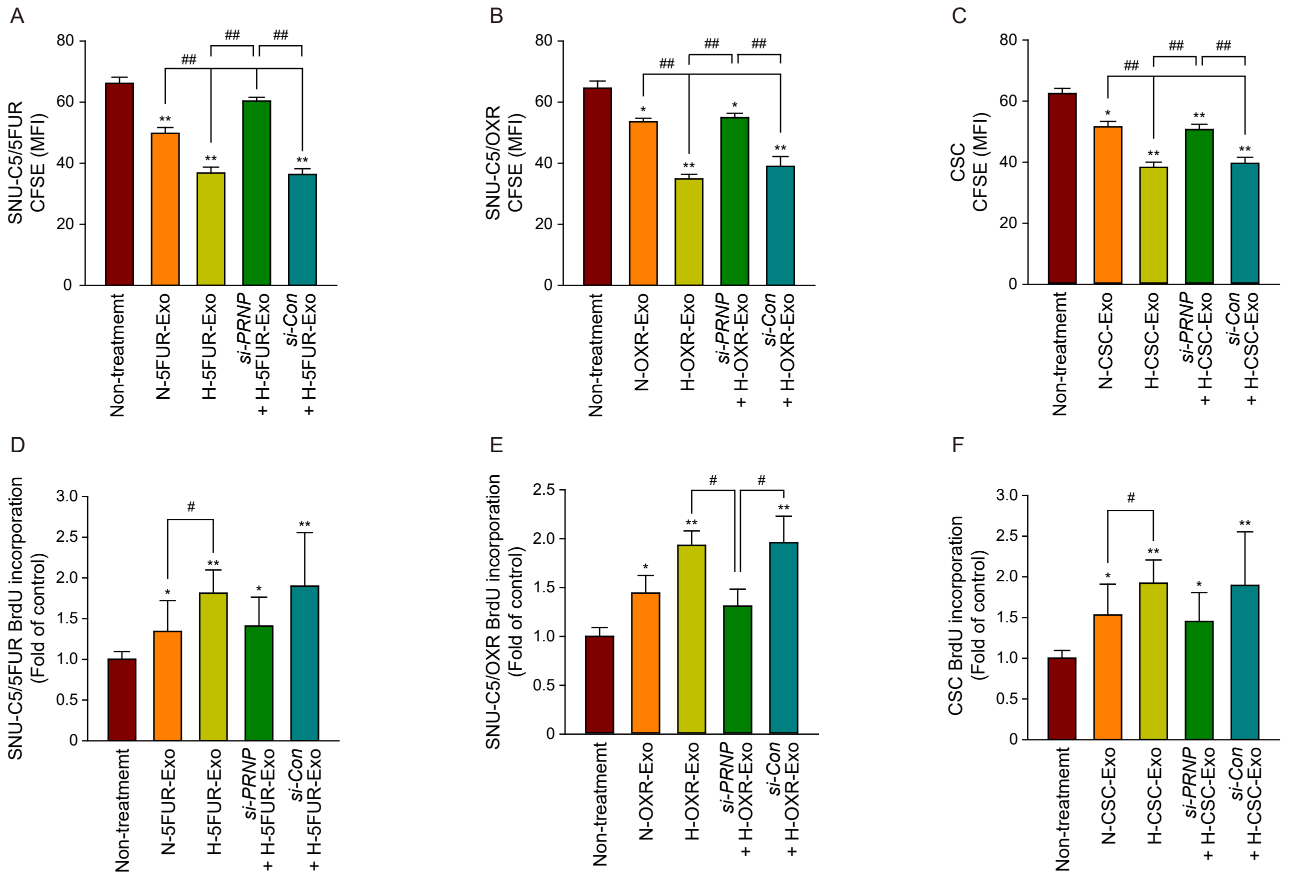

Supplemental Figure 9

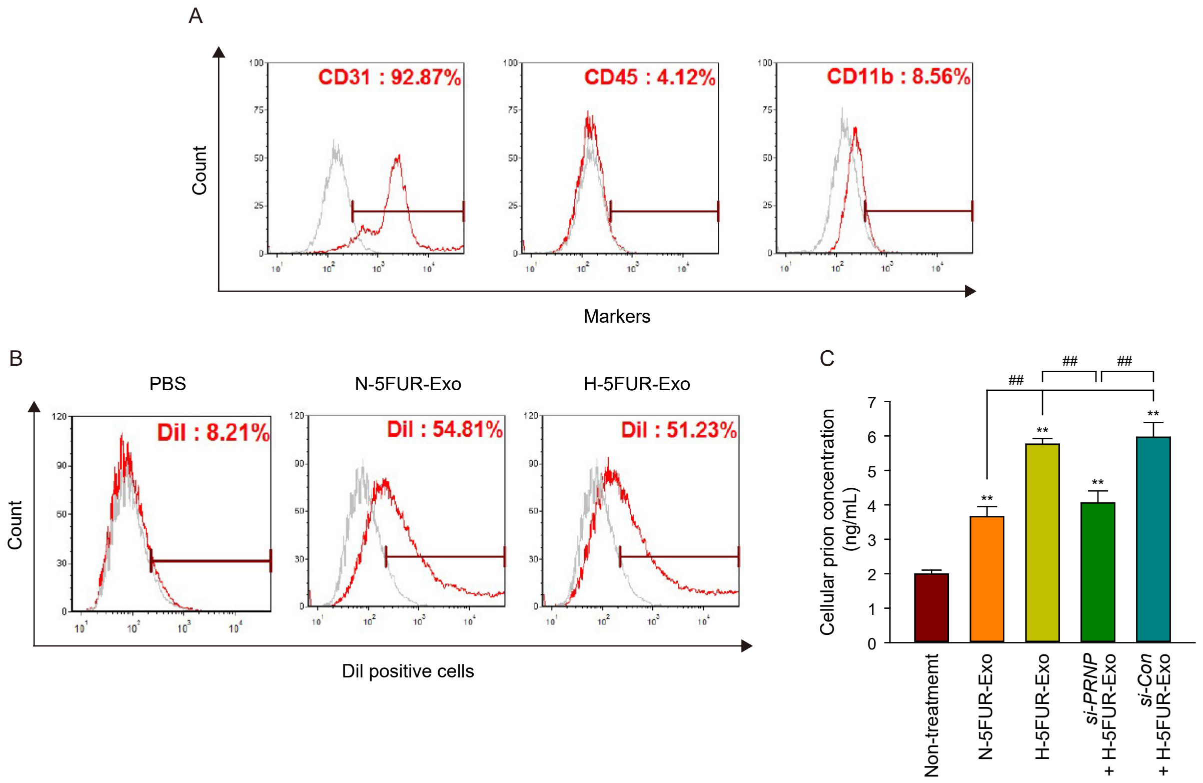

Supplemental Figure 10

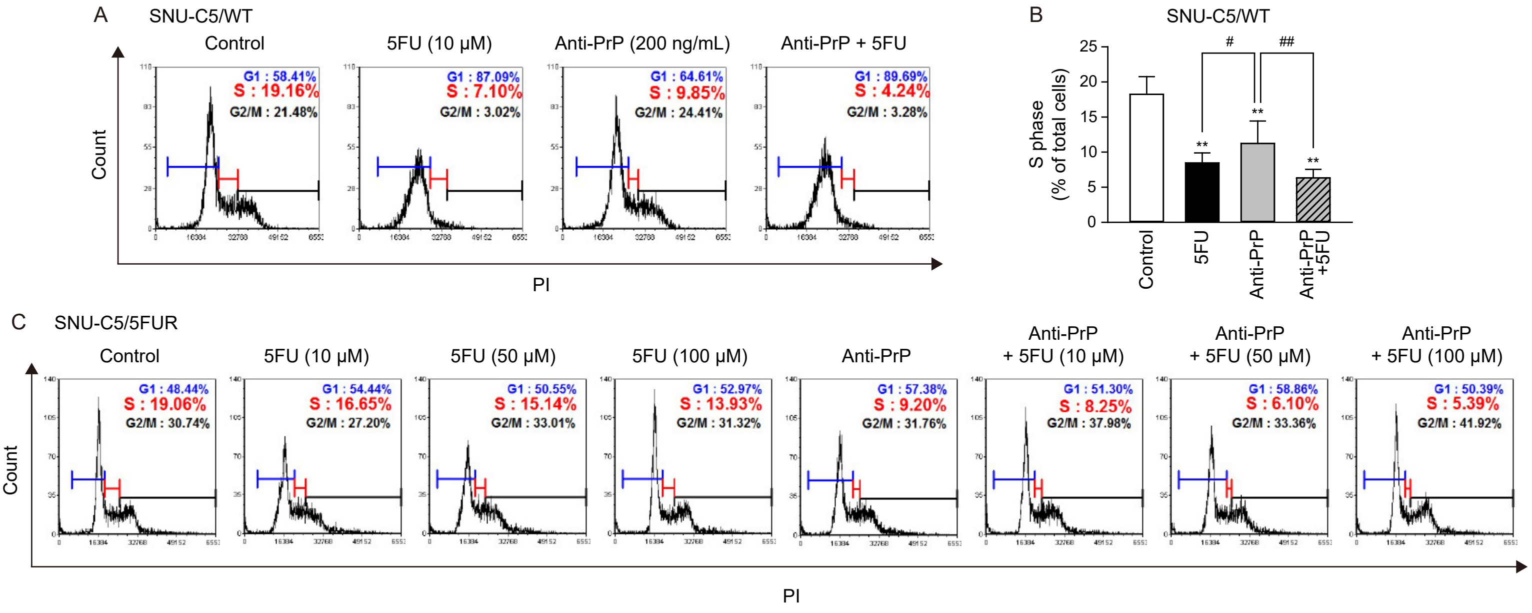

Supplemental Figure 11

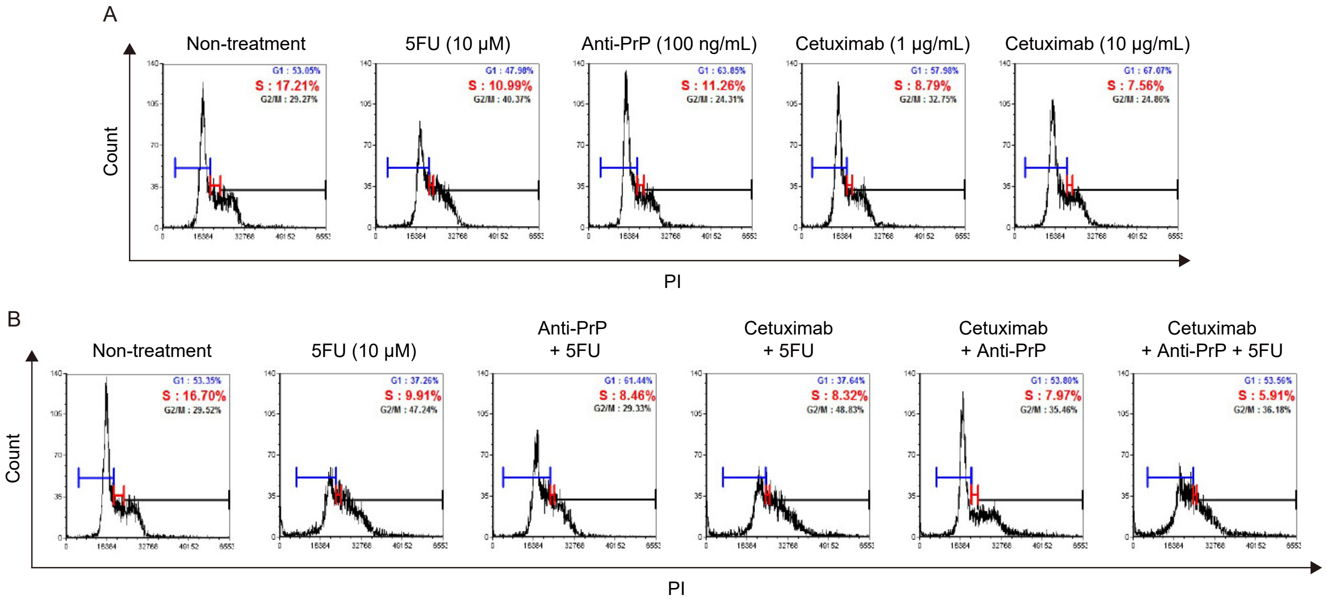

Supplemental Figure 12

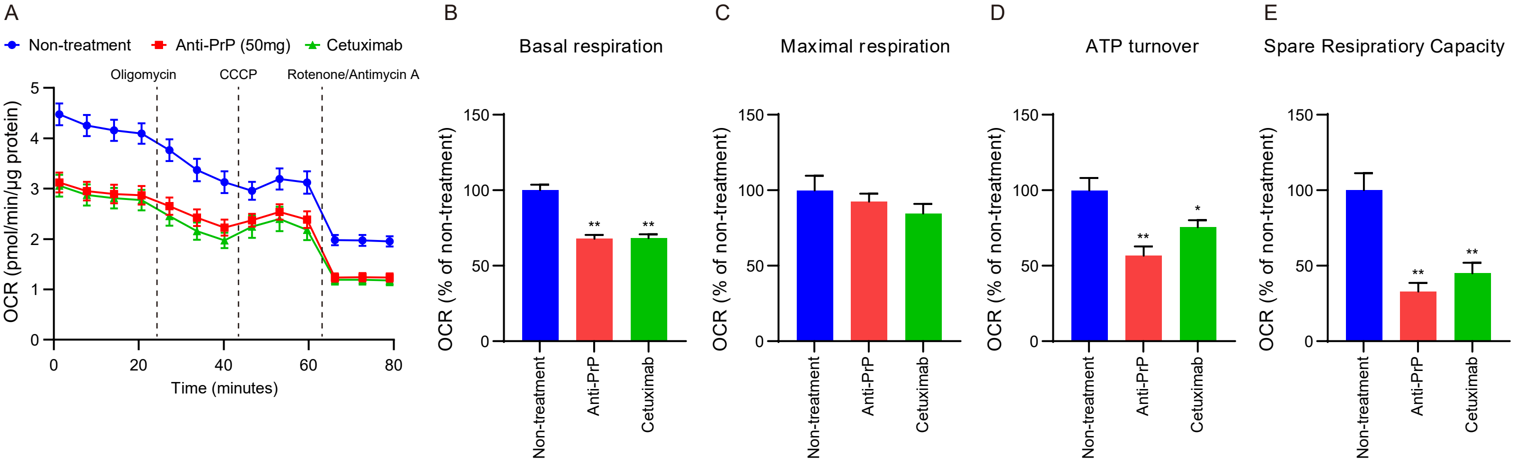

Supplemental Figure 13

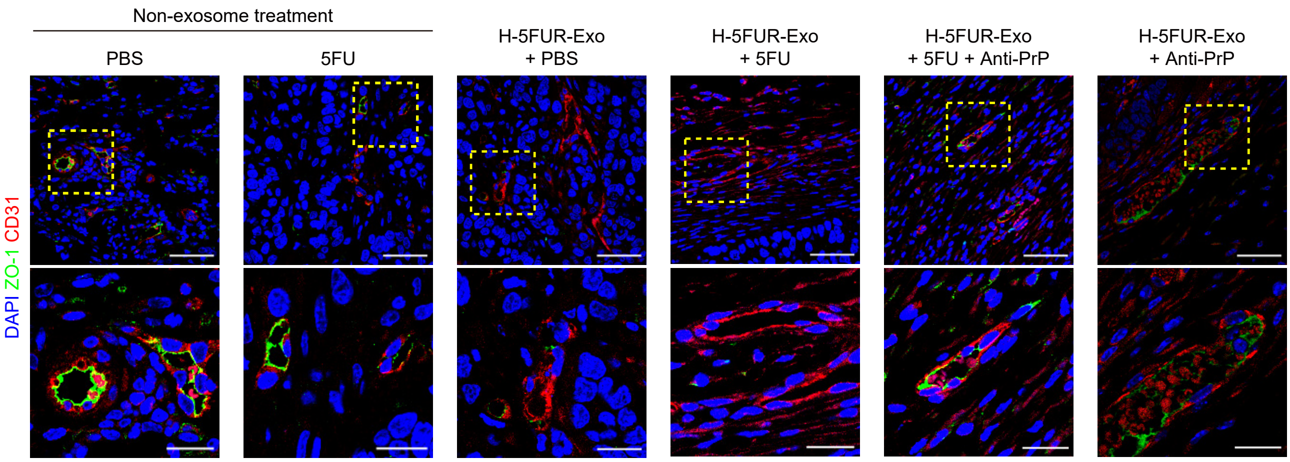

Supplemental Figure 14

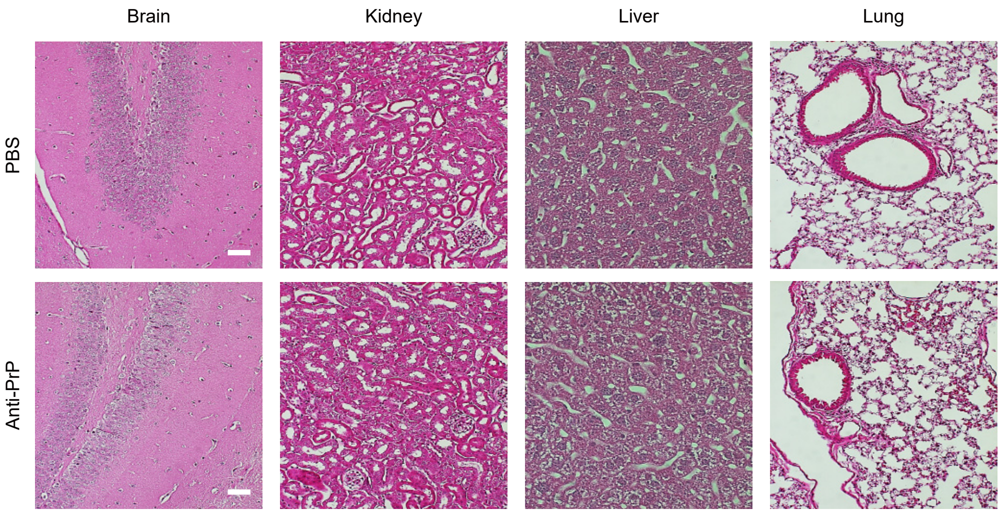

Supplement: Supplementary file 1 [file cancers-13-02144-s001.zip › cancers-1143079-supplementary.pdf]
