# Supplementary figures and images for: Prion Protein of Extracellular Vesicle Regulates the Progression of Colorectal Cancer
Source: Cancers (Basel). 2021 Apr 29;13(9):2144. doi: 10.3390/cancers13092144 (PMC8124505; doi:10.3390/cancers13092144)

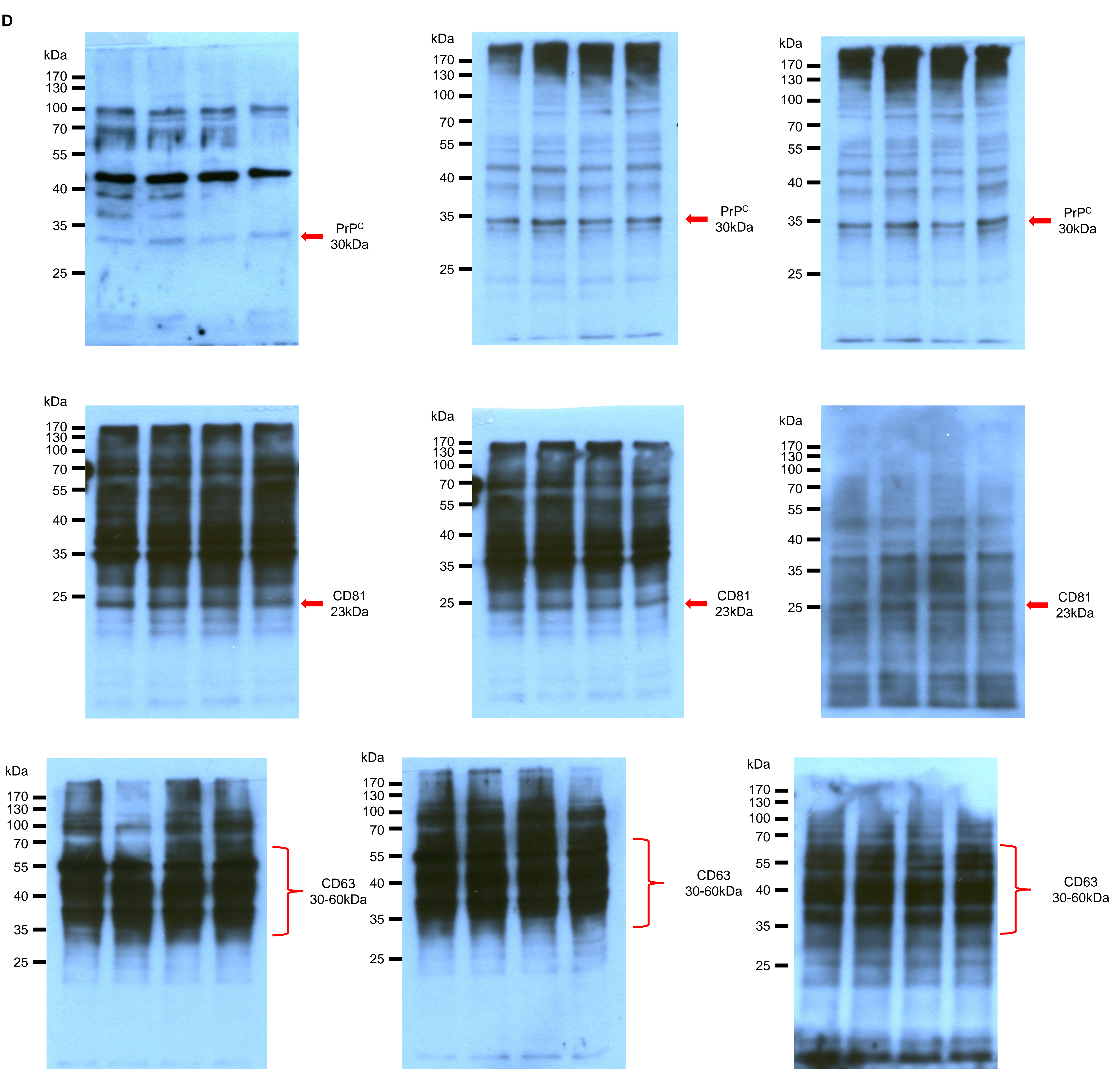

**Fig.3 raw data**

Supplement: Supplementary file 1 [file cancers-13-02144-s001.zip › cancers-1143079-original-images.pdf]
